# Ecogenomics sheds light on diverse lifestyle strategies in freshwater CPR

**DOI:** 10.1186/s40168-022-01274-3

**Published:** 2022-06-04

**Authors:** Maria-Cecilia Chiriac, Paul-Adrian Bulzu, Adrian-Stefan Andrei, Yusuke Okazaki, Shin-ichi Nakano, Markus Haber, Vinicius Silva Kavagutti, Paul Layoun, Rohit Ghai, Michaela M. Salcher

**Affiliations:** 1 Department of Aquatic Microbial Ecology, Institute of Hydrobiology, Biology Centre of the Academy of Sciences of the Czech Republic, Na Sádkách 7, 370 05 České Budějovice, Czech Republic; 2 Limnological Station, Department of Plant and Microbial Biology, University of Zurich, Kilchberg, Switzerland; 3 Bioinformatics Center, Institute for Chemical Research, Kyoto University, Gokasho, Uji, Kyoto, Japan; 4 Center of Ecological Research, Kyoto University, 2-509-3 Hirano, Otsu, Shiga Japan; 5 Department of Ecosystem Biology, Faculty of Science, University of South Bohemia, Branišovská 1760, České Budějovice, Czech Republic

**Keywords:** Patescibacteria, CPR, Freshwater lakes, Metagenomics, Genome reduction, Metabolism, Lifestyle, CARD-FISH

## Abstract

**Background:**

The increased use of metagenomics and single-cell genomics led to the discovery of organisms from phyla with no cultivated representatives and proposed new microbial lineages such as the candidate phyla radiation (CPR or Patescibacteria). These bacteria have peculiar ribosomal structures, reduced metabolic capacities, small genome, and cell sizes, and a general host-associated lifestyle was proposed for the radiation. So far, most CPR genomes were obtained from groundwaters; however, their diversity, abundance, and role in surface freshwaters is largely unexplored. Here, we attempt to close these knowledge gaps by deep metagenomic sequencing of 119 samples of 17 different freshwater lakes located in Europe and Asia. Moreover, we applied Fluorescence in situ Hybridization followed by Catalyzed Reporter Deposition (CARD-FISH) for a first visualization of distinct CPR lineages in freshwater samples.

**Results:**

A total of 174 dereplicated metagenome-assembled genomes (MAGs) of diverse CPR lineages were recovered from the investigated lakes, with a higher prevalence from hypolimnion samples (162 MAGs). They have reduced genomes (median size 1 Mbp) and were generally found in low abundances (0.02–14.36 coverage/Gb) and with estimated slow replication rates. The analysis of genomic traits and CARD-FISH results showed that the radiation is an eclectic group in terms of metabolic capabilities and potential lifestyles, ranging from what appear to be free-living lineages to host- or particle-associated groups. Although some complexes of the electron transport chain were present in the CPR MAGs, together with ion-pumping rhodopsins and heliorhodopsins, we believe that they most probably adopt a fermentative metabolism. Terminal oxidases might function in O_2_ scavenging, while heliorhodopsins could be involved in mitigation against oxidative stress.

**Conclusions:**

A high diversity of CPR MAGs was recovered, and distinct CPR lineages did not seem to be limited to lakes with specific trophic states. Their reduced metabolic capacities resemble the ones described for genomes in groundwater and animal-associated samples, apart from Gracilibacteria that possesses more complete metabolic pathways. Even though this radiation is mostly host-associated, we also observed organisms from different clades (ABY1, Paceibacteria, Saccharimonadia) that appear to be unattached to any other organisms or were associated with ‘lake snow’ particles (ABY1, Gracilibacteria), suggesting a broad range of potential life-strategies in this phylum.

Video Abstract

**Supplementary Information:**

The online version contains supplementary material available at 10.1186/s40168-022-01274-3.

## Background

Patescibacteria, also known as candidate phyla radiation (CPR) is a bacterial phylum [1] with peculiar ribosomal structures [2], reduced metabolic capacities, and small genome and cell sizes [3]. While the majority of this phylum remains uncultivated, some bacteria of the Saccharimonadia group (formally known as TM7) were successfully cultivated alongside their actinobacterial hosts [4–6] and ‘Ca. Vampirococcus lugosii’ and ‘Ca. Absconditicoccus praedator’, members of the Gracilibacteria class, were cultivated along their Gammaproteobacterial hosts [7, 8]. Another CPR bacterium (‘Ca. Sonnebornia yantaiensis’) was shown to be an endosymbiont of the protist *Paramecium bursaria* [9]. Together, these observations lead to a general assumption of an unusual host-associated, symbiotic or parasitic lifestyle for CPRs [3, 10, 11]. At this point, most CPR genomes have been recovered from groundwater samples [11–14], with only a few studies focusing on CPRs in soils [15, 16], marine systems [17, 18], boreal peatland ecosystem [19], and freshwater lakes [20, 21], although, they have been frequently reported in 16S rRNA amplicon studies [22, 23]. Because of this gap in knowledge, our study aims to present the first comprehensive investigation of CPRs in freshwater lakes on a wide scale, evaluating aspects that are still largely unknown, such as their abundance, distribution, and metabolic capacities. Additionally, we wanted to understand if freshwater CPR genomes have peculiar characteristics and whether a general host-associated lifestyle is common in the whole radiation.

Studies on CPR in freshwater lakes showed that they represent about 3–4.5% in the bathypelagic of Lake Baikal, ~ 2.5% in Lake Tanganyika and between 12 and 13% in a permafrost thaw lake in the 0.22 μm fraction [20, 21, 24]. Their ecological role is little understood, but they appear to have enzymatic resistance to O_2_ [20, 21], while being able to ferment acetate and pyruvate. Additionally, metagenome assembled genomes (MAGs) from the permafrost lake encoded many carbohydrate active enzymes, which probably have a key role in transforming complex organic matter present in freshwater lakes [21].

Previous studies hypothesized that some CPR lineages could have the capacity for independent survival [3, 10, 25], but no direct evidence exists at the moment. In order to investigate if a universal lifestyle is adopted by the whole radiation, the association of different CPRs lineages with other organisms in freshwater lakes was analyzed using multiple CARD-FISH probes. In addition, we used the representative genomes from GTDB (Genome Taxonomy Database) [1] together with the MAGs obtained in this study to compare the genome characteristics of CPRs as a whole group with known parasitic/symbiotic and free-living freshwater bacteria with the scope of finding where on this spectrum different CPR groups fit. For instance, we considered genome sizes, the number of genes, coding densities, GC content or the number of pseudogenes, aspects that evolve in different directions in host-associated vs. free-living bacteria when genome streamlining occurs [26]. The completeness of metabolic pathways and the presence of secretion systems was also checked in each genome, as the lack of the ability to synthesize amino acids, nucleotides, lipids, cofactors, or to generate ATP could indicate a dependency upon other microbes or their local environments, while some secretion systems may imply a direct interaction with a host (e.g., type III, IV, VI, and VII secretion systems) [27].

## Materials and methods

### Sample collection, DNA extraction, and metagenomic sequencing

Freshwater samples (*n* = 119) were collected from 17 lakes located in Europe and Asia (Supplementary Fig. S1, Supplementary Table S1). Details about the sampling procedures, DNA extraction and sequencing were previously published for Lakes Baikal [20], Biwa [28], Medard, Zurich, and Constance [29] as well as for Jiřická pond and the Řimov Reservoir [30]. The Ikeda samples were collected after 5 μm prefiltration on 0.22 μm Sterivex cartridge until the filter got clogged, which occurred after 2–5 L lake water were filtered. Then, DNA was extracted by PowerSoil DNA Isolation Kit (MoBio Laboratories). For the other 10 lakes, approximately 20 L of water were collected from the epi- and hypolimnion (Supplementary Table S1). The water was sequentially filtered through a 20-μm mesh plankton net to remove larger organisms, followed by 5 μm, and 0.22 μm polyethersulfone membrane filters (Millipore, Merck, Darmstadt, DE) until they got completely clogged. These filters were immersed in DNA/RNA Shield (Zymo Research, Irvine, CA, USA) and stored at – 80 ^°^C until later use. The 0.22 μm filters were cut with sterile scissors into small pieces, followed by DNA purification using the ZR Soil Microbe DNA MiniPrepTM kit (Zymo Research, Irvine, CA, USA) according to the manufacturer’s instructions. Shotgun metagenomic sequencing (2 × 151 bp) was performed using the Illumina Novaseq 6000 machine or NextSeq 500 platform.

### Data preprocessing, metagenomic assembly and binning

BBMap project tools (https://github.com/BioInfoTools/BBMap/) were used to preprocess the raw data [31]. In brief, the bbduk.sh script was used to remove poor quality reads (qtrim = rl trimq = 18), the phiX and p-Fosil2 control reads (*k* = 21 ref = vectorfile ordered cardinality), and the Illumina adaptors (*k* = 21 ref = adapterfile ordered cardinality). Preprocessed reads were assembled *de novo* with MEGAHIT v1.1.4-2 [32] using default parameters and the following selection of k-mers: 29, 49, 69, 89, 109, 119, 129, and 149. Only contigs ≥ 3 kbp were further used for binning. Quality filtered reads were mapped on the contigs to obtain mean base coverage for each contig, and hybrid binning (tetranucleotide frequencies and coverage data) was performed using MetaBAT2 with default parameters [33]. Gene prediction for all bins was performed with Prodigal v2.6.3 [34] and the taxonomy of the genes was determined by screening each gene against the genes in the GTDB r89 [1] and UniProt release 2020-02 with MMseq2 [35] (blast criteria for each protein: evalue 1e−3, similarity 10%, coverage 10%, bitscore 50) and retrieving the taxonomy of the best hit gene. Contigs that had less than 30% of the genes assigned to the dominant taxonomic class for their bin were considered contaminants and were eliminated. Viral sequences were predicted with both VirSorter [36] and Vibrant [37] tools, and contigs with > 25% of genes of viral origin were discarded. Completeness of CPR bins was assessed using a set of 43 single-copy genes (SCGs) (Supplementary Table S2) [38]. CheckM v1.0.18 [1] was run using these 43 SCGs in order to estimate bin completeness, contamination, and strain heterogeneity. Bins with > 40% completeness (based on the 43 SCGs set) and < 5% contamination were selected for further analysis (282 bins). The set of high-quality bins obtained from each lake (Supplementary Table S3) was dereplicated using dRep (average nucleotide identity (ANI) > 99%) [39], resulting in 174 representative bins (bins with highest dRep score, a metric that considers genome sizes, levels of completeness and contamination, strain-heterogeneity, N50 as well as how similar each genome is to all other genomes in their cluster) (Supplementary Table S4). These bins were classified with GTDB-Tk v1.3.0 [40] toolkit (https://ecogenomics.github.io/GTDBTk/) based on the GTDB r89. SSU rRNA gene sequences were identified in a subset of 20 million reads for each metagenome using ublast [41] and SSU-ALIGN [42] with the RDP release 11 database [43] clustered at 90% identity. The SILVA SSU database RefNR99 138 was used for taxonomic assignment [44].

### Genome annotation

Proteins were predicted with Prodigal v2.6.3, while rRNA and tRNA coding sequences were predicted using rna_hmm3 [45] and tRNAscan-SE [46] respectively. Protein annotations were performed with an in-house pipeline that uses hmmsearch [47] against collections of COG [48], TIGRFAM [49], Pfam [50] hidden Markov models (HMMs), and KOALA algorithm against a non-redundant KEGG GENES database [51]. For a protein to be considered a hit a minimum coverage of 50% for its whole length as well as the HMM model was mandatory, using an *e*-value threshold of 1e−3. Protein domains were annotated using InterProScan [52] with default parameters. Metabolic pathways were inferred from KEGG [53] and were manually examined for completeness in all MAGs (282 non-dereplicated, high quality bins) (Supplementary Table S5). Carbohydrate-active enzymes (CAZy) were searched in the MAGs using hmmscan [47] and the dbCAN CAZyme domain HMM database v10 (release date 17. 08. 2021) [54].

### Fragment recruitment, growth rate, and doubling time estimation

All 119 metagenomes from the 17 lakes were used for fragment recruitment (Supplementary Table 6). CPR MAGs were screened for rRNA gene sequences with barrnap (http://www.vicbioinformatics.com/software.barrnap.shtml) and these sequences were masked to avoid biases. From each metagenome, 20 million quality filtered reads were mapped against our MAGs using RazerS 3 (--no-gaps, --max-hits 1,000,000) [55]. The obtained number of hits was used to compute coverage per Gbp values, offering normalized abundances comparable for different MAGs and metagenomes (Supplementary Table S6). The rate of bacterial replication was estimated using the GRiD multiplex module with default options [56]. As recommended by the developers, only the forward reads from each metagenome were used for mapping and the GRiD refined values were subjected to further analysis. Doubling time for each genome was estimated with the default parameters (excluding temperature option fit) using gRodon [57].

### Bacterial lifestyle assessment

RefSeq release 81 reference genomes [58] were annotated and manually inspected for the presence of possible symbionts, parasites or commensals (for simplicity only the term symbionts will be further used). The description in the literature for each bacterium was the main criteria for including a genome in the symbionts category. Other features such as genome size, low GC%, or reduced coding density were also considered [26]. Based on this criteria, 254 genomes were classified as symbionts. A similar manual approach was performed to obtain a free-living freshwater bacteria database, checking the literature, and downloading genomes from NCBI. The final collection contained 359 free-living freshwater bacteria. Basic genome statistics, as well as functional annotations were used to compare CPRs with the free-living freshwater and symbiotic bacteria (Supplementary Tables S7-S10).

### Phylogenomic analysis

The genomes of all representative CPR species were retrieved from GTDB r89 (1031 genomes in total). The set of 43 SCGs (Supplementary Table S2) [38] was used to build a phylogenetic tree that included 1203 CPR bins and 13 other bacterial genomes as outgroup (affiliated to Deferribacteres, Fusobacteria, Spirochaetota, Aquificae, and Epsilonproteobacteria). Individual markers were aligned with PRANK (-protein +F) (Loytynoja 2014), trimmed with BMGE (-m BLOSUM62 -t AA -g 0.5 -b 5) [59] and concatenated. A maximum-likelihood tree was generated with IQ-TREE (-bb 1000, -alrt 1000) [60] with ultrafast bootstrapping and the LG + R10 evolutionary model suggested as the best model for the dataset by ModelFinder [61]. A minimum of 21 markers were required for a genome to be retained in the tree. Markers present in less than half of the bins were removed from the final multiple alignment. Based on these criteria, the phylogenetic tree was generated using 38 markers and 1196 genomes.

### Phylogenetic tree reconstruction for terminal oxidases

Gene prediction was done for all GTDB representative genomes using Prodigal v2.6.3, and the three/four subunits (COG0843, COG1622, COG1845, COG3125) of heme/copper-type cytochrome/quinol oxidase (HCO) were extracted with hmmsearch [47], followed by the generation of individual blast databases for these proteins. A local blast search was run for identifying the closest 1000 sequences of each HCO subunit from our freshwater bins. For every subunit, the BLAST results were dereplicated using MMseqs2 (easy-cluster workflow) with a minimum sequence identity of 90%. As subunit I is conserved in all HCO [62], we added sequences belonging to all phyla to this tree. To reduce the number of proteins in the alignment and tree, HCO subunit I sequences from GTDB were clustered at 70% identity (15682 initial sequences reduced to 1051). Representative sequences of this clustering step, together with those obtained through BLAST were aligned with MAFFT v7.055b [63], and the multiple alignment was trimmed with BMGE (-m BLOSUM62 -t AA -g 0.5). A Maximum-likelihood (ML) tree was generated with IQ-TREE (-bb 1000, -alrt 1000) using mtZOA + F + R10 as the best-fit evolutionary model [60, 64, 65]. As the other two/three HCO subunits are not conserved, the ML trees were generated using only the clustered top 1000 blast hits (blast *e*-value 1e−10; best evolutionary models: COG1622–LG + R6, COG1845–LG + F + R5, COG3125–mtInv + F + R6). The same approach as for HCO subunits II–IV was used for cytochrome bd-type oxidase subunits I (COG1271) and II (COG1294). Top 1000 blast hits were clustered by MMseq2 at 90% sequence similarity and were used to generate maximum likelihood trees (blast *e*-value 1e−10; best evolutionary model for both subunits was LG + F + R9).

### Phylogenetic analysis of rhodopsins

All GTDB representative CPR genomes, together with our 282 freshwater bins and recently published CPR MAGs [66] were scanned for the presence of rhodopsins. First, proteins were predicted using Prodigal v2.6.3 and then we used hmmsearch to find significant hits to rhodopsin HMMs, specifically Pfam HMMs corresponding to bac_rhodopsin and heliorhodopsin. All hits with more than 150 amino acids and *P* values < 1e−2 were selected and blasted using MMseqs against a rhodopsin database that included all known rhodopsin sequences in UniProt [67] and GTDB. The top 50 hits for each query sequence were aligned with MAFFT (--localpair –maxiterate 1000), and the multiple sequence alignment was analyzed using Polyphobius for transmembrane helix predictions [68]. Each candidate protein was checked for the number of helices (7 for canonical rhodopsins), orientation of the protein (Type I rhodopsins vs. heliorhodopsins), and the motifs for retinal binding in transmembrane helix 7 (DxxxK for Type I rhodopsins vs. SxxxK for heliorhodopsin). A number of 511 putative rhodopsin sequences were aligned with PASTA [69] and the phylogenetic tree was generated using IQ-TREE 2 with 1000 ultrafast bootstrap replicates and the LG + F + G4 evolutionary model [70].

### Probe design and fluorescence in situ hybridization followed by catalyzed reporter deposition (CARD-FISH)

Oligonucleotide probes targeting the 16S rRNA gene of different CPR lineages were designed as previously described [71]. Briefly, all CPR MAGs were screened for 16S rRNA gene sequences with barrnap (http://www.vicbioinformatics.com/software.barrnap.shtml), were aligned with the SINA web aligner [72], imported into ARB [73] and added to the reference tree of the SILVA SSU database RefNR99 138 [44] with Maximum Parsimony. Randomized Axelerated Maximum Likelihood subtrees (RAxML, 100 bootstraps, GTR-GAMMA model [74]) for different CPR lineages including 240 16S rRNA gene sequences from MAGs and closely related reference sequences from the database were computed after manual improvements of alignments. A final RAxML tree (100 bootstraps, GTR-GAMMA model) was constructed for the most promising candidates for probe design selected by high values in fragment recruitment in metagenomes and the availability of corresponding samples for FISH (Supplementary Fig. S2). Probes were designed with the tools Probe_Design and Probe_Match in ARB and all candidate probes were checked in silico for specificity and coverage in ARB and online using the TestProbe function of SILVA [44]. Competitor probes were designed for two probes that target out-group sequences by allowing 1–2 mismatches [75]. Formamide concentrations for CARD-FISH as well as mismatch and competitor analyses were estimated online with the tool MathFish [76].

For preparation of CARD-FISH samples, lake water was immediately fixed with formaldehyde (2% final concentration) at room temperature for 2 h or at 4 °C overnight. Between 5 and 10 ml of water (5 ml for eutrophic, 9–10 ml for oligotrophic lakes) were slowly filtered (100 mbar maximum pressure) onto 47 mm diameter 0.2 μm pore-sized polycarbonate filters (Millipore, Merck, Darmstadt, DE) and washed twice by filtering 5 ml of Milli-Q® water. Filters were air dried and stored at 20 ^o^C before being embedded in 0.1% agarose. Enzymatic pretreatment was done using lysozyme (10 mg/ml of lysozyme, 50 mM EDTA, and 0.1 M Tris-HCl, 60 min, 37 °C) and achromopeptidase (60 U, 1 mM NaCl, 1 mM Tris-HCl, 30 min, 37 °C), followed by an inactivation step using 0.01 M HCl for 10 min at room temperature. CARD-FISH was performed as described previously using fluorescein-labeled tyramines [77]. The newly designed probes (Supplementary Table S11, Supplementary Fig. S2) were tested with a gradient of formamide concentrations guided by MathFish predictions to achieve optimal hybridization conditions. Negative controls for unspecific binding of fluorescein and cellular peroxidases were done using the nonspecific probe NON338 [78] and the CARD reaction only, i.e., FISH was done without adding a probe to the hybridization buffer. A double hybridization with a general bacterial probe was carried out for two CPR lineages (SacA-77 and Pgri-121) that are also targeted by probe EUB I–III [79] while all other CPR lineages have > 1 mismatch with this probe. Double hybridization was done as previously described [80, 81] by using tyramides labeled with Alexa546 (probe EUBI-III) and fluorescein (probes SacA-77 and Pgri-121). All filters were counterstained with 4′,6-diamidino-2-phenylindole (DAPI) and analyzed by epifluorescence microscopy (Zeiss Imager.Z2, Carl Zeiss, Oberkochen, DE) with a colibri LED light system and filter sets for DAPI (LED module 385 nm; filter set 49; Excitation 365; beam splitter [farb teiler, FT] 395; Emission BP [Em BP] 445/50), fluorescein (LED module 475 nm; filter set 38 HE; Excitation band pass [Ex. BP] 470/40; FT 495; Em BP 525/50) and autofluorescence (LED module 567 nm; filter set 62 HE; Ex BP 370/40, 474/28, 585/35; FT 395 + 495 + 610; Em TBP 425 + 527 + long pass LP 615). In case of double hybridization, we additionally used the filter set for DsRed (LED module 567 nm; filter set 43; Excitation 545; Emission 572; FT 570). Multi-channel z-stack micrographs (9–11 z-stacks with 100 nm offset for each channel) of CARD-FISH-stained cells were recorded with an Axiocam 506 (Carl Zeiss, Oberkochen, DE) and merged to one image by orthogonal projection using the software ZEN 2.6 (Carl Zeiss, Oberkochen, DE). Cell dimensions (length and width) of individual CARD-FISH stained cells were measured on the DAPI channel with the software NIS – Elements AR 4.6 using the Annotation and Measurements tool.

## Results

### Diversity of CPRs in freshwater lakes and their general genome characteristics

**Fig. 1:**
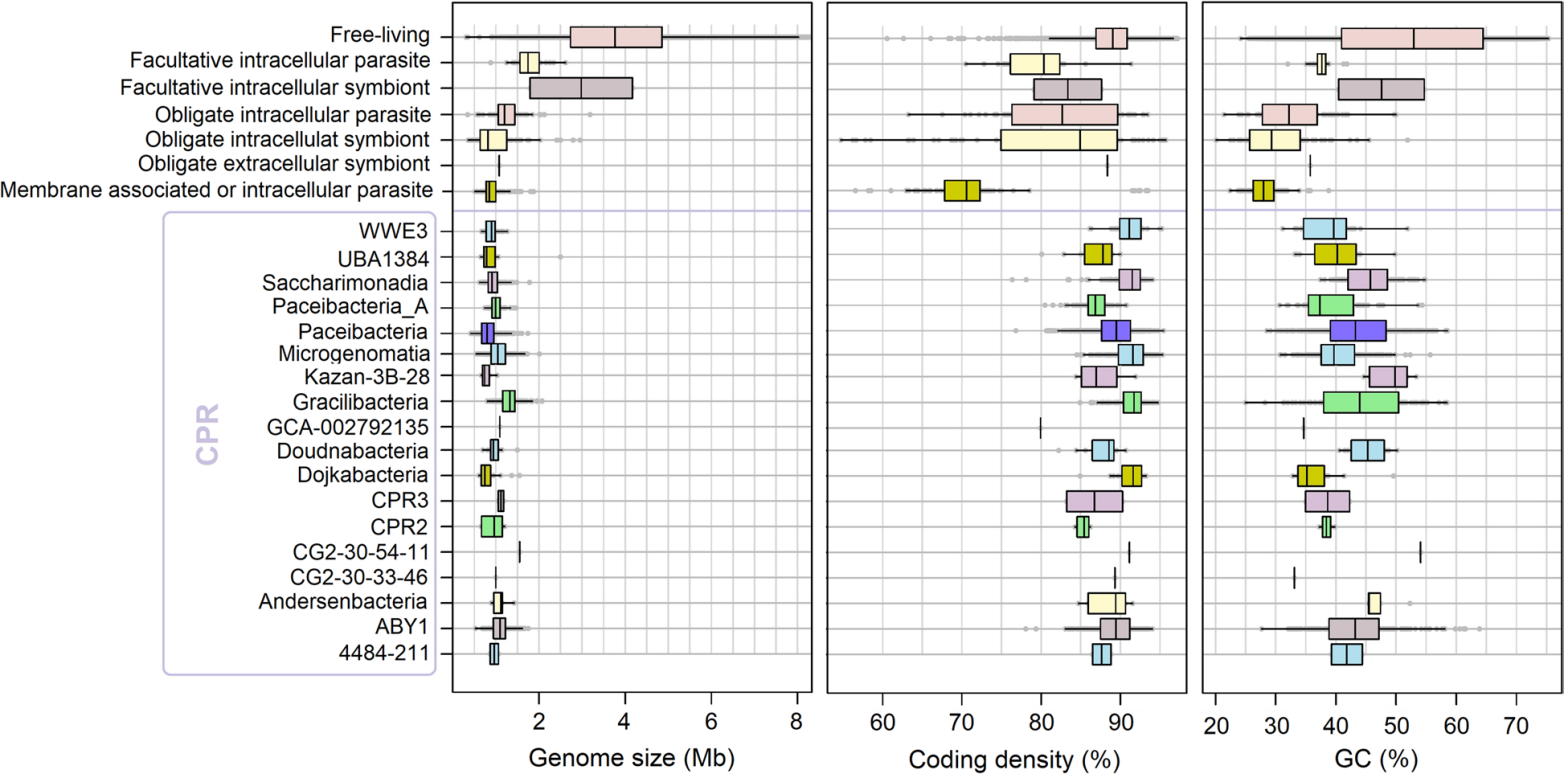
General genome characteristics for CPR classes compared to free-living bacteria and known parasites or symbionts. All representative CPR genomes from GTDB r89 were selected for this purpose together to the 282 MAGs assembled in this study. RefSeq 81 database was manually curated, and the genomes were classified in different life-strategies categories

A total of 282 CPR MAGs (> 40% completeness, < 5% contamination) from 8 classes were assembled from 119 freshwater metagenomes collected from 17 lakes. Their estimated genome sizes ranged from ~ 0.5 to 2.5 Mbp (median 1.02 Mbp, Fig. 1; assembly length ~ 0.2–1.5 Mbp, median ~ 0.63 Mbp, Supplementary Table S3). When compared to organisms with known lifestyles from RefSeq r81 (Fig. 1, Supplementary Table S7), CPRs have genome sizes and numbers of genes comparable to those of obligate intra- or extracellular symbionts/parasites. Nevertheless, their coding density (median 89.47%; range 76–95%) and GC content (median 42.04%; range 24–63%) in general resemble free-living organisms or facultative intra- or extracellular symbionts/parasites.

**Fig. 2:**
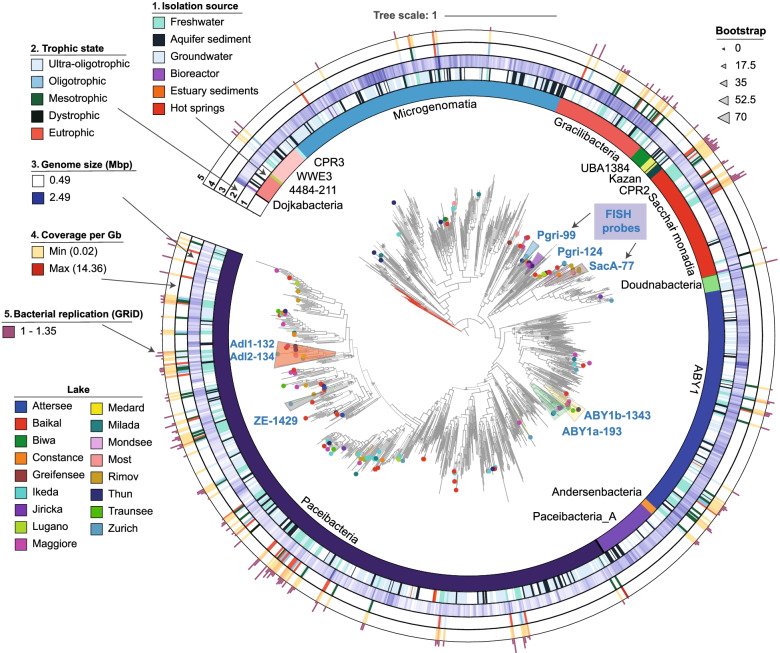
Maximum likelihood (LG + R10, general matrix and FreeRate model with 10 categories for amino acid substitution; 1000 ultrafast bootstraps) phylogeny for the CPR radiation based on 38 concatenated SCGs (Supplementary Table 2). The lake origin of each freshwater MAG obtained from this study is marked by a dot with a different color at the end of the tips. CPR classes are shown with distinct colors in the inner circle. Following annotations starting from the inner circles represent (1) the isolation source of the genome; (2) the estimated genome size; (3) the trophic state of the lake; (4) the relative abundance of each MAG in metagenomic read recruitment expressed as coverage per Gb of metagenome; (5) the GRiD values for estimation of bacterial replication rates. Probe targets for CARD-FISH visualization are indicated by different colors and the name of probes marked with light blue

A phylogeny was generated using 38 single copy genes (SCGs) [11] for 1012 representative CPR genomes retrieved from GTDB r89 together with 171 dereplicated freshwater genomes obtained in this study (Fig. 2, Supplementary Table S4). In the phylogenetic tree, no clear grouping was observed based on either the genome size, isolation source, or the trophic state of the lake. Metagenomic fragment recruitment was used to estimate the abundance of different CPR MAGs in 119 freshwater metagenomes. Coverage per Gbp values for each bin in their own metagenome varied between 0.02 and 14.36. The highest coverage was recovered for MAGs obtained in the hypolimnion of Lake Ikeda (~ 2–8.1) and the epilimnion of Lake Zurich (6.7–14.3) (Supplementary Table S6). Based on 16S rRNA gene data, Lake Ikeda and Jiricka pond had the highest percentage of CPRs (maximum values for these lakes were 10.89 and 21.05%, respectively), especially of sequences affiliated to the classes Paceibacteria, ABY1, and Microgenomatia (Supplementary Fig. S3). Gracilibacteria and Saccharimonadia appear to be only minor components of the lake communities (~ 1% abundance) according to the 16S rRNA gene data. Nevertheless, these quantitative results might underestimate the true abundance of CPRs in our samples as some free or unattached small cells could have passed through the 0.22 μm membrane filters.

Fragment recruitment analyses suggested MAGs were generally specific to the lake of origin, although a few exceptions to this rule were observed. Almost identical genomes from the hypolimnion of Lake Thun and both epi- and hypolimnion of Lake Traunsee were recovered in the hypolimnion of Lake Maggiore (ANI value of 98.56–98.83%), lakes located at relatively short distances (~ 400 km between Lake Traunsee and Maggiore and ~ 100 km between Lake Thun and Maggiore), while MAGs assembled from Lake Baikal were also found in Lake Biwa (ANI value of 99.53%, ~ 3000 km distance between lakes) (Supplementary Tables S6 and S12).

**Fig. 3:**
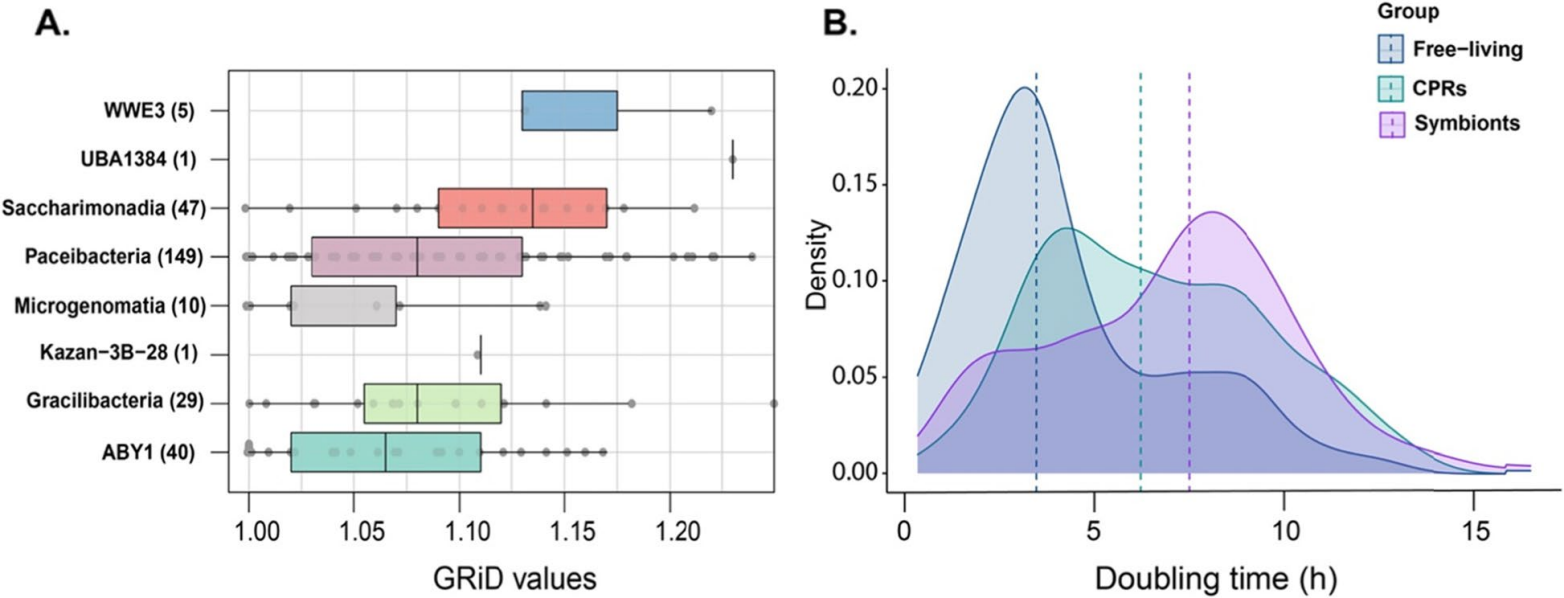
**A** Boxplot of GRiD values for freshwater CPRs according to their classes. **B** Growth rate estimations for freshwater CPRs obtained in this study and for our genome collections of free-living organisms and symbionts. Doubling time was predicted using gRodon and the median value for each group is represented by a vertical line

Genome replication rates in freshwater CPRs, based on ori/ter values provided by GRiD, varied between 1 and 1.35 (Supplementary Table S13, Fig. 3A), indicating slow growth or even stagnation at the time of sampling. Doubling time estimates for CPRs, symbionts, and free-living bacteria followed a binomial distribution (Fig. 3B), with the main peak in case of CPRs and free-living microbes around 4 h, whereas symbionts were predicted to replicate slower (median ~ 7.5 h).

### Assessment of different lifestyles in the CPR group

**Fig. 4:**
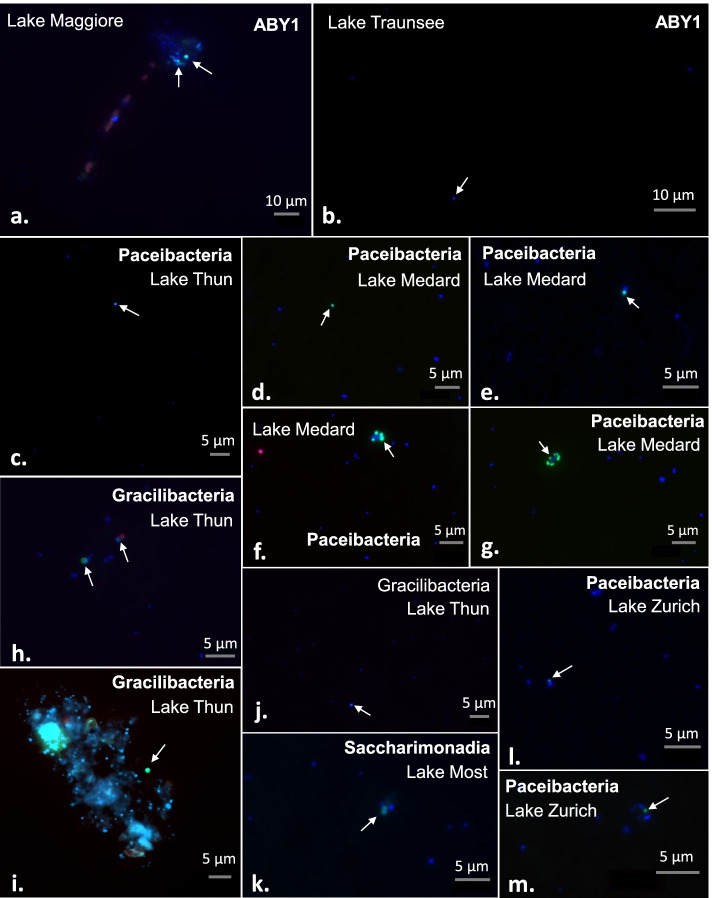
CARD-FISH imaging of different CPR clades. The panels show an overlap of the probe (green), DAPI (blue) and autofluorescence (red) signals. **a**, **b** ABY1 members from the GWF2-40-263 and UBA9934 families stained with 2 distinct probes (probe ABY1a-193 and ABY1b-1343). **c**–**f** Paceibacteria proposed genus GWA1-54-10 visualized with 2 probes (adl1-132 and adl2-134). **h**, **i** Bacteria affiliated to Gracilibacteria proposed genus 2-02-FULL-48-14 are observed using the Pgri-99 probe. **j** Organisms of the family level group LOWO2-01-FULL-3 (Gracilibacteria) stained with the Pgri-124 probe. **k** Members of the Saccharimonadia uncultivated family UBA10212 were observed using the SacA-77 probe. **l**, **m** Paceibacteria family level group UBA11359 was detected at the surface of other prokaryotes using the ZE-1429 probe

Natural abundances and visualization of most CPR lineages has remained elusive till now. To amend this, we designed eight FISH probes targeting different CPR lineages from four classes: ABY1 (2 probes), Paceibacteria (3 probes), Gracilibacteria (2 probes), and Saccharimonadia (1 probe) (Fig. 2, Supplementary Table S11, Supplementary Fig. S2). This approach enabled the visualization of distinct CPR groups and brought into light new evidence about their potential life strategies (Fig. 4; Supplementary Figs. S4–S11). A double hybridization with probe EUB I–III [79, 80] was possible only for 2 probes (SacA-77 and Pgri-121) as all other CPR lineages have > 1 mismatch to this general bacterial probe. Both double hybridizations resulted in a staining with both fluorochromes (Supplementary Figs. S12–S13), proving that the targeted CPR lineages are indeed bacteria. Negative controls using a nonspecific probe (NON338 [78]) and the CARD reaction without probe resulted in low, unspecific background signals, but no obvious staining of cells (Supplementary Figs. S14–S15). The very low abundances of individual CPR lineages targeted by our probes (Supplementary Fig. S3) did not allow a precise quantification, however, multiple images were recorded, and cells could be sized (between 20 and 71 cells per probe, Supplementary Table S11). CPRs were generally small in size (0.36–0.70 μm length, 0.30–0.59 μm width, Supplementary Table S11, Supplementary Fig. S16), but in the same range as genome-streamlined free-living freshwater microbes like ‘*Ca*. Nanopelagicales’ (0.33–0.50 μm length, 0.24–0.30 μm width [82]) or ‘*Ca*. Fonsibacter’ (0.38 μm length, 0.27 μm width [83]). However, the observed cell sizes could be partially a result of filter size (0.2 μm) used for this approach, as smaller cells might have passed through.

#### ABY1

While being similar in terms of metabolic potential (data not shown), the two targeted ABY1 families of the same order (SG8-24) showed slightly different lifestyle preferences (Fig. 4a, b; Supplementary Figs. S4 and S5). Members of candidate family UBA9934, targeted by probe ABY1b-1343 (Fig. 2, Supplementary Figs. S2 and S5) were found exclusively unattached to other cells, while members of family GWF2-40-263 (probe ABY1a-193, (Supplementary Fig. S4) targeting several MAGs from this study and one MAG by Anantharaman et al. [11] were either free-living or attached to so-called ‘lake snow’, aggregates of living or decomposing microorganisms kept together by extracellular polymeric substances, and an important source of organic matter [84, 85]. Cell sizes for the 2 families were very similar, averaging 0.52 ± 0.12 μm in length and 0.46 ± 0.12 μm in width for GWF2-40-263 and 0.52 ± 0.14 μm by 0.45 ± 0.14 μm in case of UBA9934 (Supplementary Table S11, Supplementary Fig. S16).

#### Paceibacteria

Representatives of the candidate family UBA11359 (probe ZE-1429) were identified as very small cocci (average size 0.35 ± 0.08 μm by 0.30 ± 0.06 μm), consistently associated with other larger prokaryotes (Fig. 4l, m, Supplementary Fig. S11). Members of the genus GWA1-54-10 (order UBA9983_A; family UBA2163; aka. ‘*Ca*. Alderbacteria’) were visualized with two CARD-FISH probes (Adl1-132 and Adl2-134, Supplementary Table S11) and they were shown to be also small (average cell sizes 0.67 ± 0.2 μm by 0.59 ± 0.18 μm for Adl1-132 and 0.37 ± 0.1 μm by 0.31 ± 0.09 μm for Adl2-134). All microbes targeted by probe Adl1-132 appear to be free-living (Fig. 4c, d, Supplementary Fig. S6). Only one free-living representative was observed with probe Adl2-134 (Supplementary Fig. S7A, B), while all other cells targeted by this probe were associated with hosts with up to 10 small GWA1-54-10 cells surrounding a large prokaryotic cell (Fig. 4e–g).

#### Gracilibacteria

Gracilibacteria family LOWO2-01-FULL-3 (order UBA1369), targeted by probe Pgri-124 (average cell sizes 0.48 ± 0.13 μm by 0.43 ± 0.12 μm), was associated with various small prokaryotes and picocyanobacteria (Fig. 4j, Supplementary Fig. S9) and therefore was not limited to a definite host. Another Gracilibacteria family (2-02-FULL-48-14) of the same order targeted by probe Pgri-99 (Supplementary Fig. S8) had cell sizes of 0.49 ± 0.12 μm by 0.45 ± 0.12 μm. They were likewise associated with small prokaryotes and cyanobacteria (Fig. 4h) but were also found in close vicinity to ‘lake snow’ particles (Fig. 4i).

#### Saccharimonadia

Members of the family UBA10212, targeted by probe SacA-77, were observed as diplococci (Fig. 4k, Supplementary Fig. S10). Although their genomes are highly reduced and they lack important metabolic pathways for survival (Supplementary Table S5), they were not always associated with other cells (Supplementary Fig. S4). They were observed either as elongated (on average 0.70 ± 0.07 μm by 0.41 ± 0.05 μm) or approximately round (average sizes 0.49 ± 0.08 μm by 0.43 ± 0.08 μm) cells.

### Metabolic capabilities in freshwater CPR groups

**Fig. 5:**
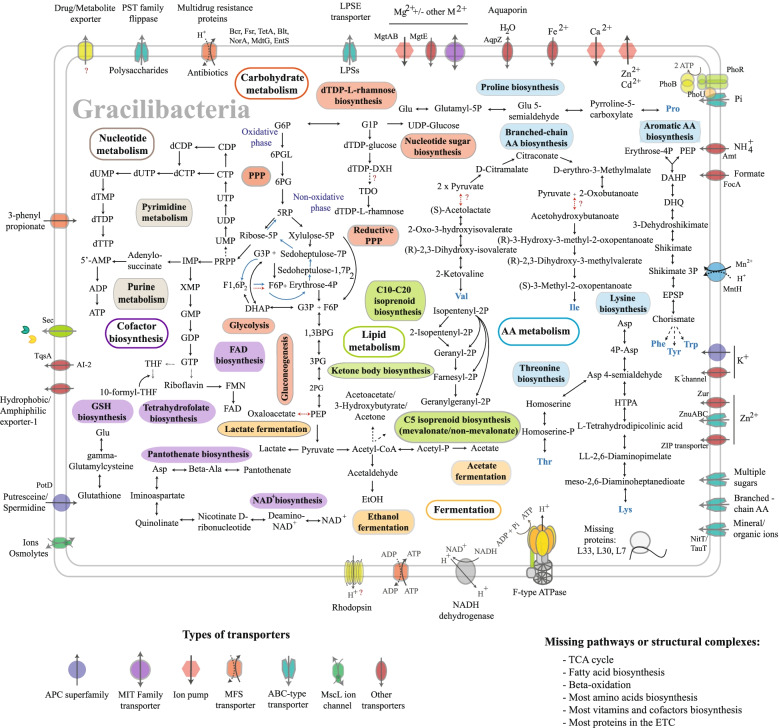
Metabolic map reconstruction for the Gracilibacteria class. Abbreviations for transporters: ABC—ATP-binding cassette, APC—amino acid-polyamine-organocation, MIT—metal ion transporter, MFS—major facilitator superfamily, MscL—large conductance mechanosensitive ion channel, PSTE—polysaccharide transporter. Abbreviations for compounds: 1,3BPG—1,3-bisphosphoglycerate, 2PG—2-phosphoglycerate, 3PG—3-phosphoglycerate, 5RP—ribulose 5-phosphate, 6PG—6-phosphogluconate, 6PGL—6-phoshoglucono lactone, DAHP—2-Dehydro-3-deoxy-D-arabino-heptonate 7-phosphate, DHAP—dihydroxyacetone phosphate, DHQ—3-Dehydroquinate, dTDP—deoxythymidine diphosphate, dTDP-DXH—dTDP-6-deoxy-D-xylo-4-hexulose, EPSP—5-enolpyruvylshikimate-3-phosphate, F1,6P2—fructose 1,6-bisphosphate, F6P—fructose 6-phosphate, FAD—flavin adenine dinucleotide, FMN—flavin mononucleotide, G3P—glyceraldehyde 3-phosphate, G6P—glucose 6-phosphate, HTPA—(2S,4S)-4-Hydroxy-2,3,4,5-tetrahydrodipicolinic acid, IMP—inosine monophosphate, LPS—lipopolysaccharides, NAD^+^—nicotinamide adenine dinucleotide, P—phosphate, PEP—phosphoenolpyruvate, PPi—pyrophosphate, PPRP—phosphoribosyl pyrophosphate, TDO—dTDP-4-oxo-L-rhamnose, THF—tetrahydrofolate, TXN—thioredoxin, TXN-S-S—thioredoxin disulfide, XMP—xanthosine monophosphate. Pathways or structural complexes: ETC—Electric transport chain, TCA—Tricarboxylic acid

In terms of average gene composition among metabolic pathways, Gracilibacteria form a cluster with free-living bacteria, while all other CPR groups possess a much more depleted metabolic repertoire and are similar to known symbionts (Supplementary Fig. S17). Gracilibacteria that were visualized by CARD-FISH (order UBA1369, formerly known as Perigrinibacteria) encode the core 3-carbon compound module of glycolysis, the genes for nucleotide sugar biosynthesis, pentose phosphate pathway (PPP), parts of the Calvin cycle, and the biosynthesis of phosphoribosyl diphosphate (PRPP) that is required to produce both purines and pyrimidines (Fig. 5). The capacity for beta-oxidation of fatty-acids and the pyruvate carboxylase are missing in all Gracilibacteria genomes along with the genes required for the synthesis of cofactors (biotin and thiamine) involved in these functions, biotin and thiamine. The genes for NAD^+^ and THF biosynthesis are present, two cofactors involved in nucleotide synthesis. The pathways necessary to produce riboflavin, FMN, and FAD were present in both Gracilibacteria orders. The same was true for pantothenate, although the enzymes required for its conversion to acetyl-CoA were not encoded in Gracilibacteria but were present in some Microgenomatia and Paceibacteria MAGs. In Gracilibacteria, acetyl-CoA can be obtained from pyruvate through the activity of pyruvate:ferredoxin oxidoreductase. Even though NADH dehydrogenase and the F-type ATPase are encoded by Gracilibacteria, together with a putative proton-pumping rhodopsin (Supplementary Table S15), terminal oxidases were not identified.

MAGs belonging to ABY1, Gracilibacteria, Microgenomatia, Paceibacteria, and Saccharimonadia usually encode the part of the reductive pentose phosphate cycle (Calvin cycle) responsible for converting glyceraldehyde 3-phosphate (G3P) to ribulose-5P (Supplementary Table S5, Supplementary Fig. S18) which is necessary for nucleotide biosynthesis. As appears common for CPRs [3, 10, 86], the Embden-Meyerhof glycolysis pathway is also rarely complete in our freshwater MAGs with only a few exceptions in Paceibacteria. In general, 6-phosphofructokinase and glucokinase/hexokinase are missing, with only the core module involving 3-carbon compounds being completely encoded in all groups. Glycolysis can still be achieved through a metabolic loop involving the pentose phosphate pathway [38] that is encoded in all classes (Supplementary Table S5). All genes encoding the enzymes involved in gluconeogenesis were present in freshwater Paceibacteria, but not in the same MAGs and therefore it is difficult to conclude if the pathway is complete. On the other hand, phosphoenolpyruvate carboxykinase, and sometimes fructose-1,6-bisphosphatase, showed patchy distributions or were missing completely in the other classes making gluconeogenesis impossible. As previously reported, enzymes involved in the Krebs cycle are not encoded in freshwater CPRs, indicating a fermentative lifestyle [25, 87].

**Fig. 6:**
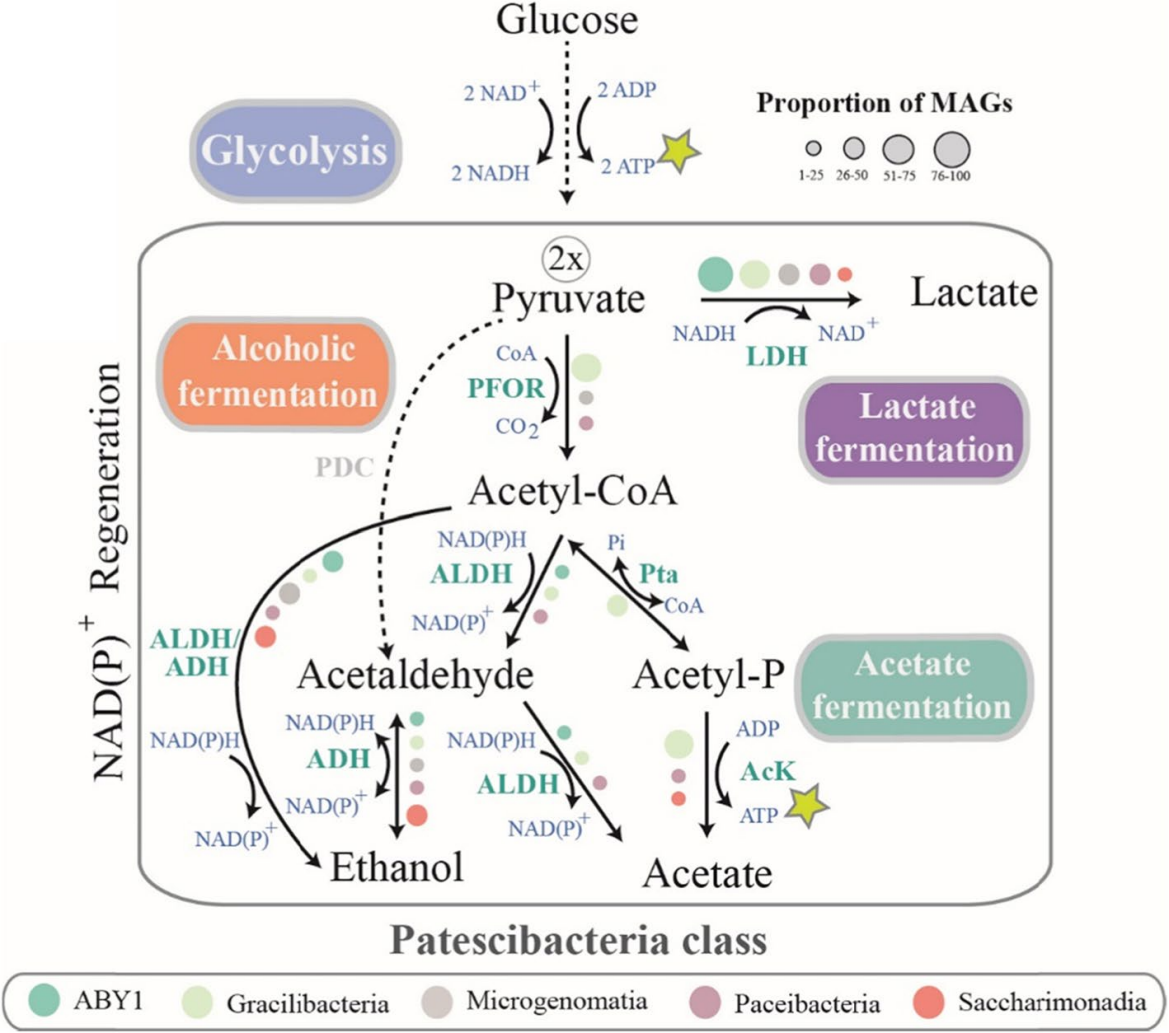
Capacity to perform certain types of fermentation in freshwater CPR. Colors mark different classes and the proportions of MAGs in each of them encoding specific enzymes is depicted by symbol size. Abbreviations for enzymes: AcK—acetate kinase, ADH—alcohol dehydrogenase, ALDH—aldehyde dehydrogenase, LDH—lactate dehydrogenase, PFOR—pyruvate:ferredoxin oxidoreductase, Pta—phosphotransacetylase

As previously described [10, 12], the capacity for fermentation is widespread in CPRs, highlighted by the prevalence of lactate dehydrogenase in ~ 50% of the freshwater MAGs assembled in this study, with the vast majority of ABY1 and Gracilibacteria possessing this gene (Fig. 6). Less common (< 30% of genomes) are the Zn-dependent and short chain alcohol dehydrogenases (ADH) that catalyze the interconversion between acetaldehyde and ethanol. Pyruvate decarboxylase, the enzyme converting pyruvate directly into acetaldehyde was not identified in our freshwater MAGs; therefore, the alcoholic fermentation could potentially occur with an additional step. Firstly, pyruvate would be converted into acetyl-CoA by pyruvate:ferredoxin oxidoreductase, and then it would be further processed by acetaldehyde dehydrogenase (ALDH). The last step involves the conversion of acetaldehyde into ethanol by alcohol dehydrogenase (ADH). Both lactic and alcoholic fermentations are coupled with the oxidation of NAD(P)H to NAD(P)+, providing a steady supply of NAD+ for powering glycolysis and the slow generation of ATP in the absence of an ETC. Although acetate kinase is common in Gracilibacteria, and to some extent in Paceibacteria and Saccharimonadia, how the acetyl-phosphate is obtained from acetyl-CoA in the last two classes is not clear, as the gene for phosphate acetyltransferase (pta) was observed only in Gracilibacteria. Nevertheless, if acetyl-P becomes available, these groups seem to be able to generate acetate by coupling this reaction with substrate level phosphorylation (Fig. 6).

All freshwater CPRs were found to encode carbohydrate-active enzymes (CAZy) with an average of 15 genes per genome (Supplementary Table S16), therefore in lower amounts than previously reported in a thermokarst lake [21]. By far, the most encountered enzymes belonged to the GT4 (involved in rhamnose degradation, found in > 95% of genomes) and GT2 (polysaccharide conversion, present in > 92.5% of genomes) families, representing ~ 35% and ~ 23% from the total number of identified CAZy. Enzymes for the degradation of chitin, cellulose, and mannose were also identified, but in low abundances, ranging from 1.1 to 2.8% of total CAZy (Supplementary Table S16).

Though carbohydrate anabolism is limited, Microgenomatia and ABY1 (Supplementary Fig. S18), and as mentioned before Gracilibacteria, can perform nucleotide sugar biosynthesis. The production of dTDP-L-rhamnose, a cell envelope component, is conserved in the ABY1, Kazan-3B-28, Microgenomatia, Gracilibacteria, Paceibacteria, and Saccharimonadia classes, while the biosynthesis of ADP-L-glycero-D-manno-heptose is encoded only in Paceibacteria (Fig. 5, Supplementary Fig. S18, Supplementary Table S5). In case of our Gracilibacteria freshwater MAGs, C5 isoprenoid biosynthesis is usually performed through a mevalonate pathway typical for eukaryotes/bacteria [3, 88], with only one exception, the UBA1369 order, that uses the common methylerythritol phosphate pathway for bacteria. Other classes seem able to perform only C10–C20 isoprenoid biosynthesis through a typical bacterial pathway (Fig. 5, Supplementary Table S5, Supplementary Fig. S18). No ability whatsoever for the synthesis or beta-oxidation of fatty acids was detected in any group, thus the way in which CPRs produce their cellular membranes remains enigmatic.

It was previously hypothesized that CPR might enable phage infection as a somewhat risky source of nucleotides [3]. By analyzing over 1300 high-quality CPR genomes, CRISPR-Cas systems were detected in 1.68–6.36% (mean = 3.89%) of the MAGs in classes with > 50 genome representatives (for a detailed report about phage defense mechanisms see Additional file 1 and Supplementary Table S14), which puts them in a low range for bacteria [89]. An alternative source for nucleotides is the uptake of free DNA from the environment. We identified competence-related DNA transformation transporters (ComEA/ComEC) in most of our MAGs, providing a stable mechanism for DNA uptake [7, 90]. Moreover, inosine monophosphate (IMP) can be synthesized from phosphoribosyl diphosphate by Gracilibacteria, Microgenomatia, and Paceibacteria. Further processing of IMP to ATP occurs in ABY1, Gracilibacteria, Microgenomatia, and Paceibacteria, but the synthesis of GTP is not completely encoded in Microgenomatia which lack guanylate kinase. Microgenomatia MAGs seem to possess all genes for uridine monophosphate (UMP) biosynthesis, but not the other CPR classes. In contrast, the ability to turn UMP into CTP is widespread in most CPR groups, as well as its conversion into TTP in ABY1, Gracilibacteria, and Paceibacteria. Metabolic pathways for the synthesis of amino acids are usually absent, with some exceptions. For example, some MAGs in Microgenomatia and Paceibacteria encode the genes necessary to produce serine, while threonine can be apparently synthesized by ABY1 (Supplementary Fig. S18), and possibly by Gracilibacteria and Paceibacteria. Genes for biosynthesis of lysine, proline, and tryptophan are present in Paceibacteria and Gracilibacteria, the latter also encoding pathways for other aromatic and branched-chain amino acids (Fig. 5). Histidine degradation to glutamate is encoded only in Paceibacteria. Although freshwater CPRs of the ABY1 class encode restricted biosynthesis pathways, its members seem to possess numerous importers for tyrosine, branched-chain amino acids, multiple sugars, 3-phenyl propionate, polysaccharides, as well as transporters for ions, such as Fe^2+^, Mn^2+^, Mg^2+^, Zn^2+^, and nitrogen oxides. They are also able to export heavy metals, polysaccharides, and numerous antibiotics (Fig. 4, Supplementary Fig. S18). Even though type II secretion systems (T2SS) were reported in CPRs [7, 90], we were able to detect only the presence of type II/IV secretion ATPase GspE together with the inner membrane platform protein GspL [91]. The remaining T2SS components were not detected, but general secretion (Sec) and twin-arginine-translocation Tat systems were found in our freshwater CPRs.

### Subunits of electron transport chain in freshwater CPRs

Regarding the genes involved in generating an electron transport chain (ETC), subunits of the NADH dehydrogenase (complex 1) are present in > 30% of the freshwater MAGs, being common in ABY1, Gracilibacteria, Paceibacteria, and Saccharimonadia. Also, three MAGs affiliated to Saccharimonadia (sampled from the oxygenated hypolimnions of lakes Most and Řimov, 50 m and 30 m depth) and four belonging to Paceibacteria (recovered from the oxygenated hypolimnion of lakes Thun and Ikeda, 180 m and 100 m depth) encode all subunits of cytochrome o oxidase (HCO), indicating a putative capacity for oxygenic respiration (complex IV). Additionally, five Paceibacteria MAGs recovered from the oxygenated hypolimnion of lakes Thun and Maggiore (180 m and 300 m depth, respectively) seem to have a functional cytochrome bd-type quinol oxidase.

The phylogeny of HCO subunit I, which included 1439 representative sequences, showed that CPRs probably obtained this gene horizontally from Proteobacteria (Supplementary Fig. S19), as previously proposed [92]. The closest group to CPRs in this tree belongs to Gammaproteobacteria, more specifically the orders Legionellales and Thiotrichales. Most of these organisms are facultative or obligate intracellular parasites, which might imply that the association with a host facilitated the HGT. Unexpectedly, the order Parachlamydiales of Verrucomicrobia, comprising mainly endosymbionts of free-living amoebae [93], appears to have obtained this subunit from Saccharimonadia. In our freshwater MAGs, HCO subunits are adjacent, forming an operon that was likely acquired horizontally at one time point. The phylogeny of the other HCO subunits follows the same evolutionary pattern as subunit I, with CPR sequences diverging from Proteobacteria, and Parachlamydiales sequences radiating from within Saccharimonadia (Supplementary Fig. S19). The ML trees generated for cytochrome bd-type oxidases (Supplementary Fig. S20) show that both subunits follow the same evolutionary pattern, in which the genes in CPRs appear to be transferred from a proteobacterial source, forming a cluster together with cyanobacterial sequences.

### Rhodopsins occurrence in CPRs

A total of 1326 CPR genomes (1032 GTDB representative genomes, 282 freshwater CPR assembled in this study, 12 MAGs analyzed by Jaffe et al. [94]) were screened for the presence of rhodopsins. We were able to detect 115 rhodopsin sequences in 86 genomes, out of which 17 were predicted to be proton-pumping rhodopsins while the rest had a reverse orientation (N-terminal in the inside, C-terminal in the outside of the membrane [95]) and were therefore inferred to be heliorhodopsins (HeRs). Both proton-pumping rhodopsins and HeRs were identified predominantly in Saccharimonadia. HeRs had 80 occurrences in Saccharimonadia, 7 in Dojkabacteria, 3 in ABY1 and less in other classes, while we identified 15 proton-pumping rhodopsin sequences in Saccharimonadia, 1 in Gracilibacteria and another 1 in Paceibacteria (Supplementary Table S15). While checking for the conserved lysine in the transmembrane helix 7 that is required for retinal binding in HeRs, we observed that the most common motifs were SLVAK, SLIAK, and SFVAK, the interchangeable amino acids belonging to the same group of compounds with hydrophobic side chains (Supplementary Table S15).

## Discussion

### Genome reduction in CPRs

One of the features of CPRs that caught the attention of the scientific community was their reduced genome sizes, cell sizes and metabolic capacities [10]. Though the mechanisms of genome reduction are different for free-living vs. symbiotic bacteria, genome reduction comes with a dependency upon other organisms: either a host, a co-symbiont, or in the establishment of a consortium with other microbes [26]. In free-living organisms, genome reduction could be an advantage especially in oligotrophic environments such as the pelagial of lakes, as it lowers the energy requirement for survival and reproduction [26]. In symbionts, gene loss occurs as a consequence of a protected and stable environment, rich in nutrients required for growth [96]. Our freshwater CPR genome sizes resembled the values reported previously for free-living streamlined bacteria [97], symbionts [98], and other CPRs [10, 38]. The higher coding density observed in CPRs (~ 76–95%, median 89%) compared to symbionts (54–95%, median ~ 76%) (Fig. 1, Supplementary Tables S8 and S10) indicates a reduced number of pseudogenes or recent gene loss, one of the traits for early stages of symbiosis and parasitism [98]. However, the observed coding densities in CPRs (Fig. 1) are still below the observed coding-densities in free-living streamlined microbes where values of > 95% are common, e.g., in freshwater ‘Ca. Nanopelagicales’ [82] or ‘Ca. Methylopumilus’ [99], suggesting that not all Patescibacteria are in the final stages of genome streamlining [3]. In some free-living and host associated bacteria, the reduction in GC% content was associated with a loss of 6-O-methylguanine-DNA methyltransferase (adaB gene), among other DNA repair genes [26]. The adaB gene is still common in our Paceibacteria and Gracilibacteria bins, but not in the other groups (data not shown). This is probably not sufficient to explain the variability in genome size and GC% values, but their drop might offer a selective advantage in phosphorus and nitrogen depleted environments such as oligotrophic and ultra-oligotrophic lakes [97, 100]. Thus, general genomic features suggest that CPRs combine characteristics of both symbiotic and free-living bacteria.

### CPRs occurrence and diversity in freshwater lakes

In the phylogenetic tree, some loose clusters were formed for MAGs belonging to Paceibacteria, ABY1, and Saccharimonadia that seem to prefer more eutrophic lakes. However, they were closely related to genomes isolated from groundwater or ultra-oligotrophic lakes (Fig. 2), suggesting that they might be more dependent on their host or co-occurring microbes than the environmental conditions [26]. Most of these MAGs belonged to the Paceibacteria class, as was also the case for Lake Baikal [20], Lake Alinen Mustajärvi [101], and a permafrost thaw lake [21], followed by Saccharimonadia and ABY1 (Supplementary Table S3). Paceibacteria, ABY1, and Microgenomatia were found in relatively high abundance in their lake of origin according to the 16S rRNA gene abundance data, while Gracilibacteria and Saccharimonadia were observed in lower proportions (~ 1% abundance) (Supplementary Fig. S3). Even at lower abundances, CPR members might still be meaningful contributors to the ecosystem as the importance of the rare microbial biosphere in nutrient cycling and organic matter breakdown is starting to be recognized [21, 102]. Genome replication results derived from GRiD (1–1.35, Fig. 3A) were similar to the values obtained for Patescibacteria in groundwaters [56], which could imply that they replicate only in certain conditions, for example when they manage to get attached to a host cell [103]. Doubling time estimation by gRodon is just tentative (Fig. 3B), as it performs poorly for slow growing organisms or for those with atypical effective population sizes, such as parasites [57]. Although CPRs have a clear peak at ~ 4 h, implying that at least a proportion of them are able to duplicate relatively fast in optimal conditions, the median is approximately at 6 h, which simply means that the doubling time cannot be predicted accurately and that the organisms are probably slow growers [57].

### CPR observations in natural environments and their interaction with hosts

Though a symbiotic lifestyle was previously assumed for this bacterial radiation, several studies hypothesized about the capacity for independent survival at least in some groups [3, 10, 13, 25]. Our CARD-FISH results indicate that a wide variety of lifestyles could exist (free-living, attached to lake snow, host-associated) among different CPR lineages (Fig. 4, Supplementary Figs. S4–S11). For example, in a single genus affiliated to Paceibacteria, we observed both free-living and host-associated cells (FISH probes Adl1-132 and Adl2-134, Supplementary Figs. S6 and S7A, B). Another peculiar observation was a group of free-living Saccharimonadia (TM7) because previous work showed them invariably in association with other bacteria [5, 6]. It is possible though that the free-living cells are facultative epibionts in search of a suitable host or they were loosely attached to host cells and got separated during sample preparation.

The relationship between parasites/symbionts and their host or the environment is carefully modulated through protein secretion. In case an association is formed between two species, secretion systems must include mechanisms to translocate secreted proteins (effectors and toxins) across the plasma membrane of the host [104]. The inner membrane platform proteins of the T2SS (GspM, GspF, GspC) were not identified, which implies that they are either too divergent or absent in Patescibacteria (Supplementary Table S5). Typically, the pseudopilus in Gram-negative bacteria is composed of five pseudopilins (GspG-K), out of which CPRs seem to encode only GspG and two of the minor pseudopilins (GspH and GspI). Furthermore, none of the outer-membrane complex proteins were identified, such as GspD (forming the secretin pore) and GspS, which questions the functional ability of T2SS. Patescibacteria were predicted to be Gram-positive [105], therefore, it is quite peculiar for them to encode parts of the inner membrane and periplasmic T2SS. General secretion (Sec) and twin-arginine-translocation Tat systems are universal in bacteria, both being used to export proteins in an unfolded state [104]. All CPR classes seem to have members that encode the complete Sec membrane complex (SecD-G, SecY) as well as the cytoplasmic SecA, which hydrolyses ATP to drive proteins translocation (Supplementary Table S5) and PrsA, a membrane-associated lipoprotein that was proven to be essential for protein secretion in *Bacillus subtilis* [106, 107]. Additionally, YidC protein is also common, mediating the membrane insertion of Sec proteins [108]. Tat translocation was identified in Microgenomatia, Paceibacteria, and Saccharimonadia, being composed of TatA (the major pore-forming subunit) and TatC (the subunit involved in recognizing the targeted proteins for secretion) proteins. Bacterial secretion systems known to directly interact with the host cell (type III, IV, VI, and VII secretion systems) were not detected, even though at least some CPRs are clearly associated with Actinobacteria [4, 5, 109] and Proteobacteria [7, 90]. Also, as it was apparent from our work, members of two Gracilibacteria families associated with cyanobacteria, although they did not seem to be restricted to them as hosts (Fig. 4, Supplementary Figs. 8 and 9). Therefore, the mode of interaction between symbiotic/parasitic CPRs and their hosts is still unknown, though some electron microscopy evidence points towards interaction through pili-like structures [3, 66].

### Energy and carbon sources in freshwater CPRs

The absence of respiration was reported to be a common feature of CPRs, making them reliant on fermentation for energy conservation [2]. Indeed, the complete set of five complex structures similar to the mitochondrial ETC was never present in any of our MAGs. Nevertheless, subunits of different respiratory complexes (complex I–NADH dehydrogenase, complex IV–terminal oxidases) are present in many freshwater CPRs and a complete F-type ATPase is usually the norm (Supplementary Table S5). The encoded oxidases form an operon in freshwater CPRs and were probably obtained as a result of a HGT event. These enzymes differ in their affinity for oxygen; compared to HCOs, cytochrome bd oxidases have a higher affinity and may allow cells to respire O_2_ even when its concentration is very low [110]. Even though we found MAGs from the same lake and lake layers encoding different terminal oxidases, it was observed that cytochrome bd-type oxidase was more often encountered at lower O_2_ concentrations (Supplementary Fig. S21). Interestingly, a group of phylogenetically closely related Saccharimonadia MAGs encoded only the F1 subunit of ATPase, lacking any other genes for the synthesis of respiratory oxygen reductases, which probably restrict them to fermentation. The almost universal presence of ATPase in CPRs together with some parts of the ETC, and the absence of any genes for respiratory oxygen reductases in the absence of functional ATPase, makes sense only in case CPRs can generate a proton motive force (PMF). It was hypothesized that the PMF might be generated using yet undescribed protein systems, unusual combinations of proteins (e.g., ferredoxins, rubredoxins) [7, 90], or maybe even rhodopsins (see the Discussion below; Supplementary Table S15). The presence of terminal oxidases in some CPRs is somewhat striking as it implies an oxygenic metabolism. Although it cannot be certain, it was proposed that the lack of other ETC components indicates an O_2_ scavenging function rather than that of energy production [25].

Interestingly, at least some Gracilibacteria seem to be able to directly import ATP due to the presence of the ATP/ADP translocase, a common transporter primarily in obligate intracellular bacteria and plant plastids [111, 112]. These transporters mediate the import of host ATP across the bacterial cell membrane, which otherwise would not be permeable for such a large and charged compound [111]. This might indicate that at least in some episymbiotic/parasitic CPR groups, the host itself or the local environment could be the direct source of ATP required for survival.

The presence of different CAZy in CPRs suggest for the capacity of degradation of both simple and complex carbon substrates, contributing to the turnover of organic matter. The capacity for rhamnose degradation is by far the most common of all (Supplementary Table S16). Rhamnose is a widespread constituent of cell walls in plants and algae, and it can become an abundant sugar in lake ecosystems, especially in the profundal zone, making it a stable monosaccharide source [113]. CAZy involved in chitin, cellulose and mannose degradation were also encoded by our freshwater CPRs, indicating for a role in decomposition and fermentation of organic matter. In any case, the capacity for fermentation is widespread in all freshwater CPR groups, allowing a slow but steady supply of ATP and the regeneration of oxidized NAD(P)^+^. Fermentation products, such as lactate, alcohol and acetate could be secreted in the environment, supporting both aerobic and anaerobic microorganisms [10], and accomplishing another key ecological role in freshwaters. Genes required for glycerolipids (GT28) and polysaccharide conversion (GT2) were also identified, representing maybe important mechanisms for obtaining these compounds in the absence of *de novo* biosynthetic pathways [21].

### Putative rhodopsins roles in CPRs

**Fig. 7:**
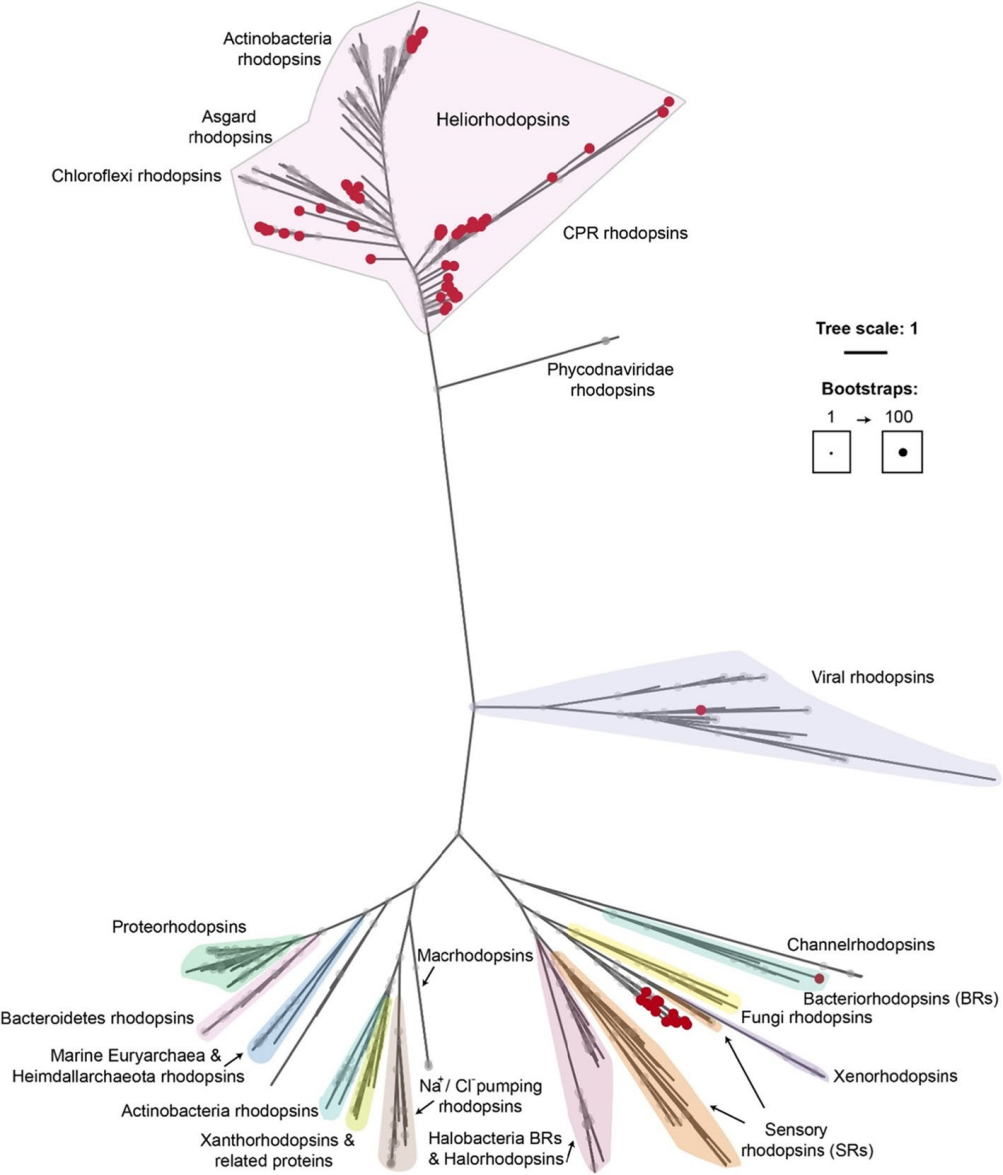
Maximum likelihood (LG + F + G4, general matrix with empirical codon frequencies counted from data and discrete Gamma model with 4 rate categories) phylogeny for rhodopsins. CPR rhodopsins are marked with a red dot at the end of the tips. A total of 511 sequences were used to generate the tree (alignment length 792), including a database of 392 proteins from known rhodopsin families

Type I rhodopsins [114] can generate a PMF or ion gradient, which could be used for energy production. As some Saccharimonadia MAGs obtained from freshwater lake samples in Sweden and Finland, as well as from glacial surface ice in Greenland [94] encoded genes annotated with low confidence as bacteriorhodopsin, it was hypothesized that they might encode protein pumping rhodopsins (Supplementary Table S15). Bins that were investigated by Jaffe et al. [94] form a freshwater cluster in our rhodopsin phylogeny in between Sensory Rhodopsin I (SRI) and II (SRII) (Fig. 7), but we were unable to find any of the proteins involved in the signal transduction from SRI/II in the near genome context [115, 116] (Supplementary Fig. S22). Therefore, these could indeed be ion-pumping rhodopsins, indicating a photoheterotrophic lifestyle in some Saccharimonadia members, as recently suggested [117].

Interestingly, the same freshwater Saccharimonadia MAGs encode also heliorhodopsins (HeRs), a recently discovered group of rhodopsins with a peculiar, reversed orientation (cytoplasmic N-terminus, extracellular C-terminus) [95]. HeRs were also found in other Saccharimonadia, as well as in ABY1, Microgenomatia, Dojkabacteria, Kazan-3B-28, UBA1384, CPR2, and CPR3 groups (Supplementary Table 15). CPR HeRs appear to form new clades in the phylogenetic tree (Fig. 7). With the use of strand-specific transcriptomics and genomic context analysis, Bulzu et al. [118] proposed that HeRs have a critical role in protecting especially monoderms against light-induced oxidative stress through the increased transcription of glyoxylases, glutaredoxins, peroxiredoxins, and catalases. While glyoxylases were present in only few freshwater CPR bins, the other enzymes are encountered in almost all MAGs. Hence, in the context of genome streamlining, protection against oxidative stress is a feature highly conserved in CPRs and at least in some cases it might be linked with the HeRs function as a sensory rhodopsin.

## Conclusion

This study presents new insights about CPRs diversity, distribution, and physiology in an under-explored habitat and on a large scale, namely 119 metagenomic samples collected from 17 freshwater lakes located in central Europe and Asia. Though in low abundance and with low replication rates, various CPR MAGs were consistently found in these lakes, with no apparent preference for the trophic state of the biome. By employing CARD-FISH, we were able to visualize several CPR lineages for the first time and the evidence suggests the existence of different life strategies in this radiation. We found likely free-living or dispersing forms of several CPRs lineages, CPRs attached to ‘lake snow’ particles and host-associated CPRs. Freshwater CPRs showed reduced metabolic capacities, similar to groundwater ones, with the exception of Gracilibacteria which seem to possess more complete metabolic pathways. Even though some MAGs encode parts of the ETC, the organisms are most probably fermenters, providing lactate and acetate for other microorganisms in the ecosystem. The presence of heliorhodopsins together with oxidative stress mitigating enzymes in CPRs indicates that protection against oxidative stress is still a conserved feature in these reduced genomes. Overall, this study brought forward new information about Patescibacteria, a still enigmatic bacterial group, and helped us expand the picture about their occurrence and life strategies to freshwater lakes.

## Supplementary Information

**Additional file 1.** Identification of phage defense mechanisms in CPRs.

**Additional file 2: Supplementary Figure S1.** Geographic location of the 17 freshwater lakes sampled in this study. Trophic state of each lake is marked by a different color. **Supplementary Figure S2.** Randomized Axelerated Maximum Likelihood (RAxML) tree of 16S rRNA genes of different classes of Patescibacteria. The tree was split in two subtrees with collapsed branches for better visibility. The 16S rRNA gene sequences recovered from MAGs generated in this study are shown in bold. Targets for eight newly designed oligonucleotide probes for CARD-FISH are indicated with different colors. Bootstrap values (100 bootstraps) are indicated as differently sized circles, the tree scale is given on top. Asterisks indicate sequences not targeted by probes pgri-99 and ABY1b-1343, # indicate one outgroup hit of probe adl2-134. **Supplementary Figure S3.** Abundance (%) of 16S rRNA gene sequences affiliated to different classes in CPR. Samples that had < 1% of total abundance in any CPR class were not added to the plot. **Supplementary Figure S4.** Epifluorescence microscopy images of ABY1 class members using the ABY1a-193 probe. Panels labeled with a. are the overlap of the probe (green), DAPI (blue) and autofluorescence (red) signal. The rest of the panels represent individual signals. **Supplementary Figure S5.** Epifluorescence microscopy images of ABY1 class members using the ABY1b-1343 probe. Panels labeled with a. are the overlap of the probe (green), DAPI (blue) and autofluorescence (red) signal. The rest of the panels represent individual signals. **Supplementary Figure S6.** Epifluorescence microscopy images of Paceibacteria class members using the Adl1-132 probe. Panels labeled with a. are the overlap of the probe (green), DAPI (blue) and autofluorescence (red) signal. The rest of the panels represent individual signals. **Supplementary Figure S7A.** Epifluorescence microscopy images of Paceibacteria class members using the Adl1-134 probe. Panels labeled with a. are the overlap of the probe (green), DAPI (blue) and autofluorescence (red) signal. The rest of the panels represent individual signals. **Supplementary Figure S7B.** Epifluorescence microscopy images of Paceibacteria class members using the Adl1-134 probe. Panels labeled with a. are the overlap of the probe (green), DAPI (blue) and autofluorescence (red) signal. The rest of the panels represent individual signals. **Supplementary Figure S8.** Epifluorescence microscopy images of Gracilibacteria class members using the Pgri-99 probe. Panels labeled with a. are the overlap of the probe (green), DAPI (blue) and autofluorescence (red) signal. The rest of the panels represent individual signals. **Supplementary Figure S9.** Epifluorescence microscopy images of Gracilibacteria class members using the Pgri-124 probe. Panels labeled with a. are the overlap of the probe (green), DAPI (blue) and autofluorescence (red) signal. The rest of the panels represent individual signals. **Supplementary Figure S10.** Epifluorescence microscopy images of Saccharimonadia class members using the SacA-77 probe. Panels labeled with a. are the overlap of the probe (green), DAPI (blue) and autofluorescence (red) signal. The rest of the panels represent individual signals. **Supplementary Figure S11.** Epifluorescence microscopy images of Paceibacteria class members using the ZE-1429 probe. Panels labeled with a. are the overlap of the probe (green), DAPI (blue) and autofluorescence (red) signal. The rest of the panels represent individual signals. **Supplementary Figure S12.** Positive control for the Saccharibacteria class members performed using the SacA-77 and EUB I-III probes. Panels labeled with a. are the overlap of DAPI (blue), the Sac-77 probe (green), the EUB I-III probe (orange) and autofluorescence (red) signal. The rest of the panels represent individual signals. **Supplementary Figure S13.** Positive control for the Gracilibacteria class members performed using the Pgri-124 and EUB I-III probes. Panels labeled with a. are the overlap of DAPI (blue), the Pgri-124 probe (green), the EUB I-III probe (orange) and autofluorescence (red) signal. The rest of the panels represent individual signals. **Supplementary Figure S14a.** Negative controls for filters used for FISH from different lakes. Panels labeled with a. are the overlap of DAPI (blue), the NON338 probe (green) and autofluorescence (red) signal. The rest of the panels represent individual signals. **Supplementary Figure S14b.** Negative controls for filters used for FISH from different lakes. Panels labeled with a. are the overlap of DAPI (blue), the NON338 probe (green) and autofluorescence (red) signal. The rest of the panels represent individual signals. **Supplementary Figure S15a.** Negative controls for filters used for FISH from different lakes. Panels labeled with a. are the overlap of DAPI (blue), CARD amplification (green) and autofluorescence (red) signal. The rest of the panels represent individual signals. **Supplementary Figure S15b.** Negative controls for filters used for FISH from different lakes. Panels labeled with a. are the overlap of DAPI (blue), CARD amplification (green) and autofluorescence (red) signal. The rest of the panels represent individual signals. **Supplementary Figure S16.** Boxplots for the length and width of individual CPR cells observed with the 8 FISH probed designed and tested in this study. L – Length, W – Width. **Supplementary Figure S17.** Percentage of genes in each KEGG module encoded by CPRs, free-living freshwater bacteria and symbionts. All representative CPR genomes from GTDB (>40% completeness, <5% contamination) were included in this analysis together with our freshwater MAGS (*n* = 1288). The same quality criteria were applied for free-living (*n* = 359) and symbionts (*n* = 227). First, an average per genome was computed, followed by an average for the whole group. **Supplementary Figure S18.** Metabolic reconstruction of ABY1 class. Abbreviations for transporters: ABC – ATP-binding cassette, LPS – lipopolysaccharides, MDR – multi drug resistance, MFS – major facilitator superfamily, MIT – metal ion transporter, MscL – large conductance mechanosensitive ion channel, PST - polysaccharide transporter, RND – resistance-nodulation-division. Abbreviations for compounds: 1,3BPG – 1,3-bisphosphoglycerate, 2PG – 2-phosphoglycerate, 3PG – 3-phosphoglycerate, 5RP – ribulose 5-phosphate, 6PG – 6-phosphogluconate, 6PGL – 6-phoshoglucono lactone, DHAP – dihydroxyacetone phosphate, dTDP – deoxythymidine diphosphate, dTDP-DXH – dTDP-6-deoxy-D-xylo-4-hexulose, F1,6P2 – fructose 1,6-bisphosphate, F6P – fructose 6-phosphate, FAD – flavin adenine dinucleotide. FMN – flavin mononucleotide, G3P – glyceraldehyde 3-phosphate, G6P – glucose 6-phosphate, P – phosphate, PEP – phosphoenolpyruvate, PPi – pyrophosphate, TDO – dTDP-4-oxo-L-rhamnose, THF – tetrahydrofolate, TXN – thioredoxin, TXN-S-S - thioredoxin disulfide. **Supplementary Figure S19.** Maximum likelihood phylogeny of heme-cupper oxidase subunits I-IV. a. Phylogeny of HCO subunit I (generated using 1439 sequences, 213 amino acid alignment length trimmed at maximum 50% gaps per position – the same filtering criteria was applied for all subunits). b. Phylogeny of HCO subunit II (694 sequences, 200 amino acid alignment length). c. Phylogeny of HCO subunit III (675 sequences, 156 amino acid alignment length). d. Phylogeny of HCO subunit IV (268 sequences, 76 amino acid alignment length). Bootstrap values are indicated by circle sizes. **Supplementary Figure S20.** Maximum likelihood trees of cytochrome bd-type oxidase subunits I-II. The best selected evolutionary model was LG + F + R9 (general matrix with empirical codon frequencies counted from the data and FreeRate model with 9 categories) for both subunits. A number of 499 and 428 sequences were used for the phylogeny of subunit I and II, respectively. **Supplementary Figure S21.** Boxplots for oxygen concentrations measured in the lake strata from where CPR MAGs encoding terminal oxidases were obtained. **Supplementary Figure S22.** Genomic context of predicted rhodopsins in CPRs that form a cluster in-between sensory rhodopsin I and II. Where contig length permitted, 5 genes were checked in the proximity of rhodopsins to search for putative proteins involved in the signaling transduction.

**Additional file 3: Supplementary Table 1.** Sampling details for the analysed metagenomes. **Supplementary Table 2.** Completeness of MAGs calculated based on 43 SCGs (Anantharaman, Brown, Hug, et al. 2016). **Supplementary Table 3.** Basic statistics and taxonomy (GTDB, NCBI) for the MAGs assembled in this study. **Supplementary Table 4.** List of final, dereplicated bins and the ones of lower quatily that were collapsed into them. **Supplementary Table 5.** Annotation results of CPRs at class level. KEGG modules were manually checked for completeness after annotationg the bins using different databases (Panda, TIGR, COG, Interproscan and Pfam). **Supplementary Table 6.** Recruitment results (coverage per Gbp) for all bins versus all samples analyzed in this study. **Supplementary Table 7.** Basic statistics for Refseq 81 database, representative CPR genomes from GTDB r89 and the MAGs assembled in this study. **Supplementary Table 8.** Basic statistics for the manually curated SPC database used in this study. **Supplementary Table 9.** Basic statistics for the manually curated free-living bacteria database used in this study. **Supplementary Table 10.** Basic statistics for the representative CPRs genomes in GTDB versions r89 database. **Supplementary Table 11.** List of CPR FISH probes (their sequence, specificity and target group) and cells measurements and statistics. **Supplementary Table 12.** Average nucleotide identity (ANI) values computed between all pairs of freshwater CPR MAGs obtained in this study. **Supplementary Table 13.** GRiD results for the dereplicated bins, estimating the rate of genome replication. **Supplementary Table 14.** Phage defense systems and their occurencess in CPRs, SPCs and free-living bacteria. **Supplementary Table 15.** Predicted rhodopsins in CPRs, including all GTDB representative genomes and the282 MAGs obtained in this study. **Supplementary Table S16.** CAZys and their occurence in freshwater CPR genomes.

## Data Availability

Sequence data generated in this study have been deposited in the European Nucleotide Archive (ENA) at EMBL-EBI under project accession numbers PRJEB35640 and PRJEB35770. The genomic data that support the findings of this paper are available in FigShare (link: https://figshare.com/s/7f5c78f4949068e5492b) and under the accession numbers ERS11897645 - ERS11897891. All other relevant data supporting the findings of this study are available within the paper and its supplementary information files.

## Abbreviations

ADH: Alcohol dehydrogenase
ADP: Adenosine diphosphate
ALDH: Acetaldehyde dehydrogenase
ANI: Average nucleotide identity
ATP: Adenosine triphosphate
CARD-FISH: Fluorescence in situ Hybridization followed by Catalyzed Reporter Deposition
CPR: Candidate phyla radiation
CTP: Cytidine triphosphate
DAPI: 4′,6-diamidino-2-phenylindole
dTDP: Thymidine diphosphate
ETC: Electron transport chain
FAD: Flavin adenine dinucleotide
FISH: Fluorescence in situ hybridization
FMN: Flavin mononucleotide
G3P: Glyceraldehyde 3-phosphate
GTDB: Genome Taxonomy Database
HCO: Heme/copper-type cytochrome/quinol oxidase
HeR: Heliorhodopsin
HMM: Hidden Markov model
IMP: Inosine monophosphate
MAG: Metagenome-assembled genome
ML: Maximum-likelihood
NAD(P)H: Nicotinamide adenine dinucleotide phosphate
NAD^+^/NADH: Nicotinamide adenine dinucleotide
PMF: Proton motive force
PPP: Pentose phosphate pathway
PRPP: Phosphoribosyl diphosphate
RAxML: Randomized axelerated maximum likelihood
SCG: Single copy genes
SSU rRNA: Small subunit ribosomal ribonucleic acid
T3SS: Type three secretion system
T4SS: Type four secretion system
T6SS: Type six secretion system
T7SS: Type seven secretion system
THF: Thetrahydrofolate
TTP: Tymidine triphosphate
UMP: Uridine monophosphate
