# Supplementary material for: Ecogenomics sheds light on diverse lifestyle strategies in freshwater CPR
Source: Microbiome. 2022 Jun 4;10:84. doi: 10.1186/s40168-022-01274-3 (PMC9166423; doi:10.1186/s40168-022-01274-3)
Supplement: Supplementary file 2 — Additional file 1. Identification of phage defense mechanisms in CPRs. [file 40168_2022_1274_MOESM1_ESM.doc]

**Additional File 1**

**Defense mechanisms**

**Identification of phage defense mechanisms in CPRs**

CRISPR loci were predicted using pilercr [1] with default parameters. For other antiphage defense systems (Thoeris, Hachiman, Shedu, Gabija, Septu, Lamassu, Zorya I and II, Druantia, Wadjet, DISARM) [2-4], a collection of Pfam and COG HMMs (Supplementary Table S11) were searched in all GTDB and our new freshwater CPR genomes (n = 1313). In the case of KwaA protein, which did not have any domain annotation, we downloaded all protein sequences found in Firmicutes (Gram-positive like CPRs) from the NCBI Protein database, aligned them with mafft (--maxiterate 1000 --localpair) and generated a corresponding HMM. A similar approach was performed for BrxE and Argonaute proteins. For each identified system we performed a manual inspection to ensure that the domains/genes are in the same operon.

**Results and discussion**

Previous studies reported that the presence of CRISPR-Cas systems in about ~ 2.4% of the CPR genomes, in striking opposition with the rest of bacteria (~24.4 - 40%) and archaea (47.4%) [2, 5, 6]. Tian et al. [7] even proposed that in the absence of CRISPR-Cas, CPRs might evade phage infection by limiting the number of phage receptor protein at the cell surface, such as flagellin, pilin, CapB, CapC and TonB proteins.

By analyzing over 1300 high quality CPR genomes, CRISPR-Cas systems were detected in 1.68 – 6.36% (mean = 3.89%) of the MAGs in classes with >50 genome representatives (Additional Figure 1; Supplementary Table S11), which puts them in a low range for bacteria. While our MAGs are not complete, the total absence of CRISPR-Cas systems in some groups might imply that their reduced genomes encode more compact restriction-modification systems for viral defense [8] or that they evolved a rather constitutive defense (e.g., loss of pilus) [9]. Though some parts of type 4 pilus (T4P) are still encoded in many CPR bins, such as the ATPase assembly protein PilB, ATPase twitching motility PilT or the membrane platform components (PilO, PilM, PilN), the actual PilA protein which represents the building blocks for the pilus is encountered in ~50% of the MAGs, with the same frequency as is free-living bacteria (Additional Figure 1). Contrarily, other genes that encode for proteins that are common targets for phages, such as those for flagellin (FliC), the iron uptake protein FhuA, the membrane porin C (OmpC) or the secretion system protein YueB were completely absent in CPRs and rarely observed in symbionts (Additional Figure 1). Moreover, the absence of CRISPR-Cas systems was associated with the lack of capacity for nucleotide biosynthesis and the adoption of a symbiotic lifestyle, both characteristics being proven at least for some CPR groups [10 - 12].


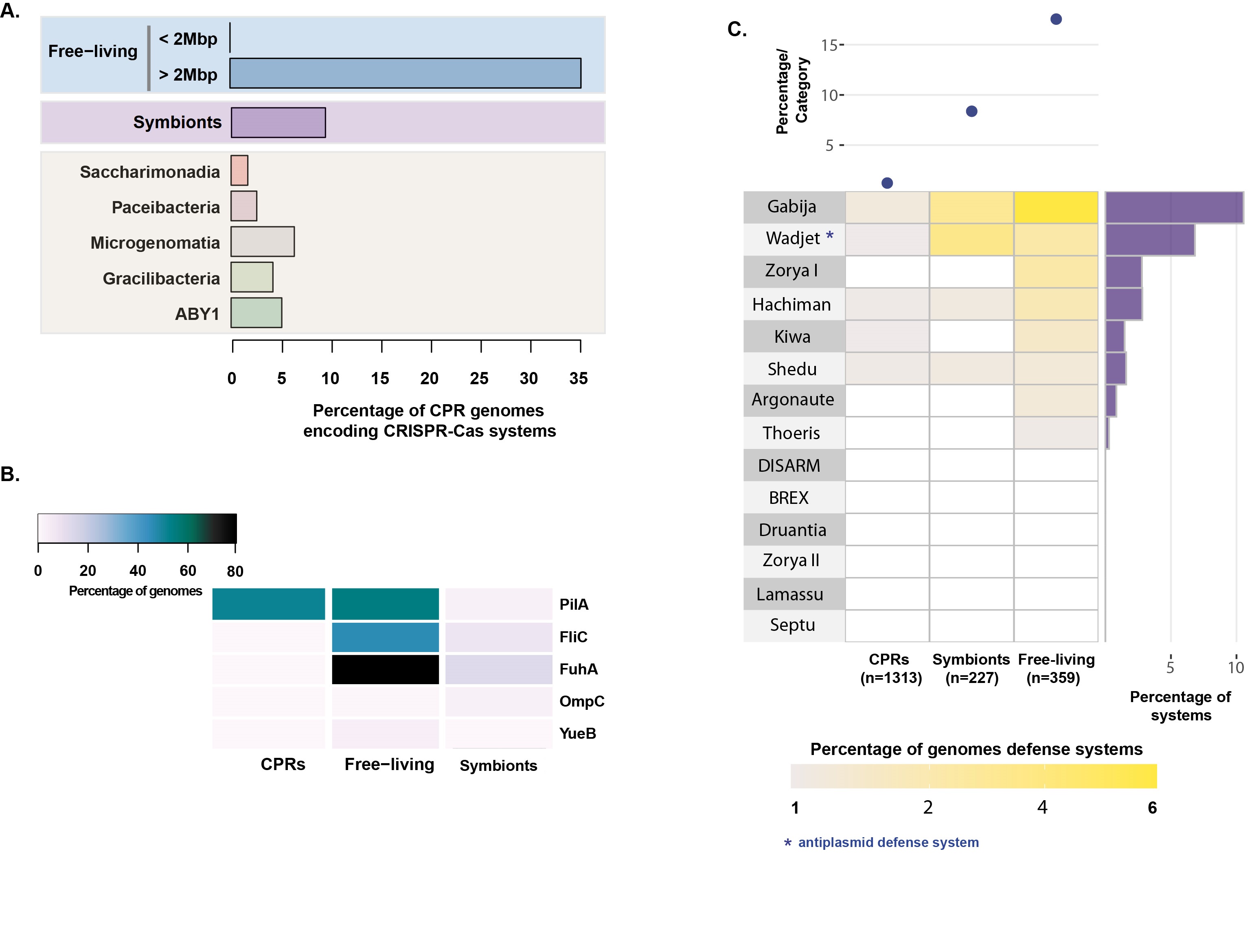
**Additional Figure 1.** **A.** Percentage of genomes in CPR classes that encode CRISPR-Cas systems**.** **B.** Occurrence of proteins that are common targets for phages in CPRs, symbionts and free-living bacteria. C. Occurrence of anti-phage defense systems in CPR, free-living freshwater bacteria, and symbionts. Each system was manually checked to ensure that genes are positioned in operons.

By looking at the presence of CRISPR-Cas systems in free-living streamlined (average genome size ~ 1.49 Mb, n = 109) versus symbiotic (average genome size ~ 1.13 Mb, n = 216) bacteria, we found that the free-living streamlined organisms do not possess any CRISPR-Cas systems, while 10% of those that have a parasitic/symbiotic lifestyle do, suggesting these parasites/symbionts might be exposed to viruses at some stage in their life-cycle as well (Additional Figure 1). Relying on constitutive defense could be therefore more advantageous for free-living bacteria and some CPR groups, as opposed to the majority of known symbiotic bacteria.

Regarding other systems located in the “defense islands”, sometimes together with CRISPR-Cas, CPRs encoded similar anti-phage systems to known symbionts, but in lower proportions (Additional Figure 1, Supplementary Table S11). Some defense mechanisms were never encountered in streamlined genomes, regardless of lifestyle, such as DISARM, BREX, Druntia, Zorya II, Lamassu and Septu. Gabija, the most abundant defense mechanism in CPRs (encoded by Paceibacteria, Saccharimonadia, ABY1, Gracilibacteria and Dojkabacteria) after CRISPR-Cas system, consists in a two gene operon (GajAB), encoding an ATPase and a helicase. Its efficiency in protection against phages was experimentally demonstrated in *Bacillus cereus*, though the mode of action remains unknown. The same is true for Kiwa (encoded by Paceibacteria), Shedu (found in Paceibacteria_A, Microgenomatia and CPR3) and Hachiman (encountered only in Paceibacteria) defense systems that were found in CPRs (Doron et al., 2018). The Wadjet operon, consisting of four genes, was identified in CPRs as well as in parasites and free-living bacteria (Additional Figure 1). Experimental evidence using *Bacillus subtilis*, ten phages targeting this organism and the episomal plasmid pHCMC05, suggests that Wadjet operon might function rather in protection against foreign plasmids than phages [2].

**References**

1. Edgar RC, Myers EW: PILER: identification and classification of genomic repeats. Bioinformatics. 2005;21 Suppl 1:i152-158.

2. Doron S, Melamed S, Ofir G, Leavitt A, Lopatina A, Keren M, Amitai G, Sorek R: Systematic discovery of antiphage defense systems in the microbial pangenome. Science. 2018; 359(6379)-eaar4120.

3. Goldfarb T, Sberro H, Weinstock E, Cohen O, Doron S, Charpak-Amikam Y, Afik S, Ofir G, Sorek R: BREX is a novel phage resistance system widespread in microbial genomes. EMBO J. 2015;34(2):169-83.

4. Ofir G, Melamed S, Sberro H, Mukamel Z, Silverman S, Yaakov G, Doron S, Sorek R: DISARM is a widespread bacterial defence system with broad anti-phage activities. Nat Microbiol. 2018;3(1):90-8.

5. Chen LX, Al-Shayeb B, Meheust R, Li WJ, Doudna JA, Banfield JF: Candidate Phyla Radiation Roizmanbacteria From Hot Springs Have Novel and Unexpectedly Abundant CRISPR-Cas Systems. Front Microbiol. 2019;10:928.

6. Godde JS, Bickerton A: The repetitive DNA elements called CRISPRs and their associated genes: evidence of horizontal transfer among prokaryotes. J Mol Evol. 2006;62(6):718-29.

7. Tian R, Ning D, He Z, Zhang P, Spencer SJ, Gao S, Shi W, Wu L, Zhang Y, Yang Y et al: Small and mighty: adaptation of superphylum Patescibacteria to groundwater environment drives their genome simplicity. Microbiome. 2020;8(1):51.

8. Burstein D, Sun CL, Brown CT, Sharon I, Anantharaman K, Probst AJ, Thomas BC, Banfield JF: Major bacterial lineages are essentially devoid of CRISPR-Cas viral defence systems. Nat Commun. 2016;7:10613.

9. Westra ER, van Houte S, Oyesiku-Blakemore S, Makin B, Broniewski JM, Best A, Bondy-Denomy J, Davidson A, Boots M, Buckling A: Parasite Exposure Drives Selective Evolution of Constitutive versus Inducible Defense. Curr Biol. 2015;25(8):1043-9.

10. Cross KL, Campbell JH, Balachandran M, Campbell AG, Cooper SJ, Griffen A, Heaton M, sJoshi S, Klingeman D, Leys E et al: Targeted isolation and cultivation of uncultivated bacteria by reverse genomics. Nat Biotechnol. 2019;37(11):1314-21.

11. Castelle CJ, Brown CT, Anantharaman K, Probst AJ, Huang RH, Banfield JF: Biosynthetic capacity, metabolic variety and unusual biology in the CPR and DPANN radiations. Nat Rev Microbiol. 2018;16(10):629-45.

12. Moreira D, Zivanovic Y, López-Archilla AI, Iniesto M, López-García P: Reductive evolution and unique infection and feeding mode in the CPR predatory bacterium *Vampirococcus lugosii*. Nat Commun. 2021;12:2454.
