# Supplementary material for: Ecogenomics sheds light on diverse lifestyle strategies in freshwater CPR
Source: Microbiome. 2022 Jun 4;10:84. doi: 10.1186/s40168-022-01274-3 (PMC9166423; doi:10.1186/s40168-022-01274-3)
Supplement: Supplementary file 3 — Additional file 2: Supplementary Figure S1. Geographic location of the 17 freshwater lakes sampled in this study. Trophic state of each lake is marked by a different color. Supplementary Figure S2. Randomized Axelerated Maximum Likelihood (RAxML) tree of 16S rRNA genes of different classes of Patescibacteria. The tree was split in two subtrees with collapsed branches for better visibility. The 16S rRNA gene sequences recovered from MAGs generated in this study are shown in bold. Targets for eight newly designed oligonucleotide probes for CARD-FISH are indicated with different colors. Bootstrap values (100 bootstraps) are indicated as differently sized circles, the tree scale is given on top. Asterisks indicate sequences not targeted by probes pgri-99 and ABY1b-1343, # indicate one outgroup hit of probe adl2-134. Supplementary Figure S3. Abundance (%) of 16S rRNA gene sequences affiliated to different classes in CPR. Samples that had < 1% of total abundance in any CPR class were not added to the plot. Supplementary Figure S4. Epifluorescence microscopy images of ABY1 class members using the ABY1a-193 probe. Panels labeled with a. are the overlap of the probe (green), DAPI (blue) and autofluorescence (red) signal. The rest of the panels represent individual signals. Supplementary Figure S5. Epifluorescence microscopy images of ABY1 class members using the ABY1b-1343 probe. Panels labeled with a. are the overlap of the probe (green), DAPI (blue) and autofluorescence (red) signal. The rest of the panels represent individual signals. Supplementary Figure S6. Epifluorescence microscopy images of Paceibacteria class members using the Adl1-132 probe. Panels labeled with a. are the overlap of the probe (green), DAPI (blue) and autofluorescence (red) signal. The rest of the panels represent individual signals. Supplementary Figure S7A. Epifluorescence microscopy images of Paceibacteria class members using the Adl1-134 probe. Panels labeled with a. are the overlap of the probe [file 40168_2022_1274_MOESM2_ESM.doc]

**Supplementary Figures**

**
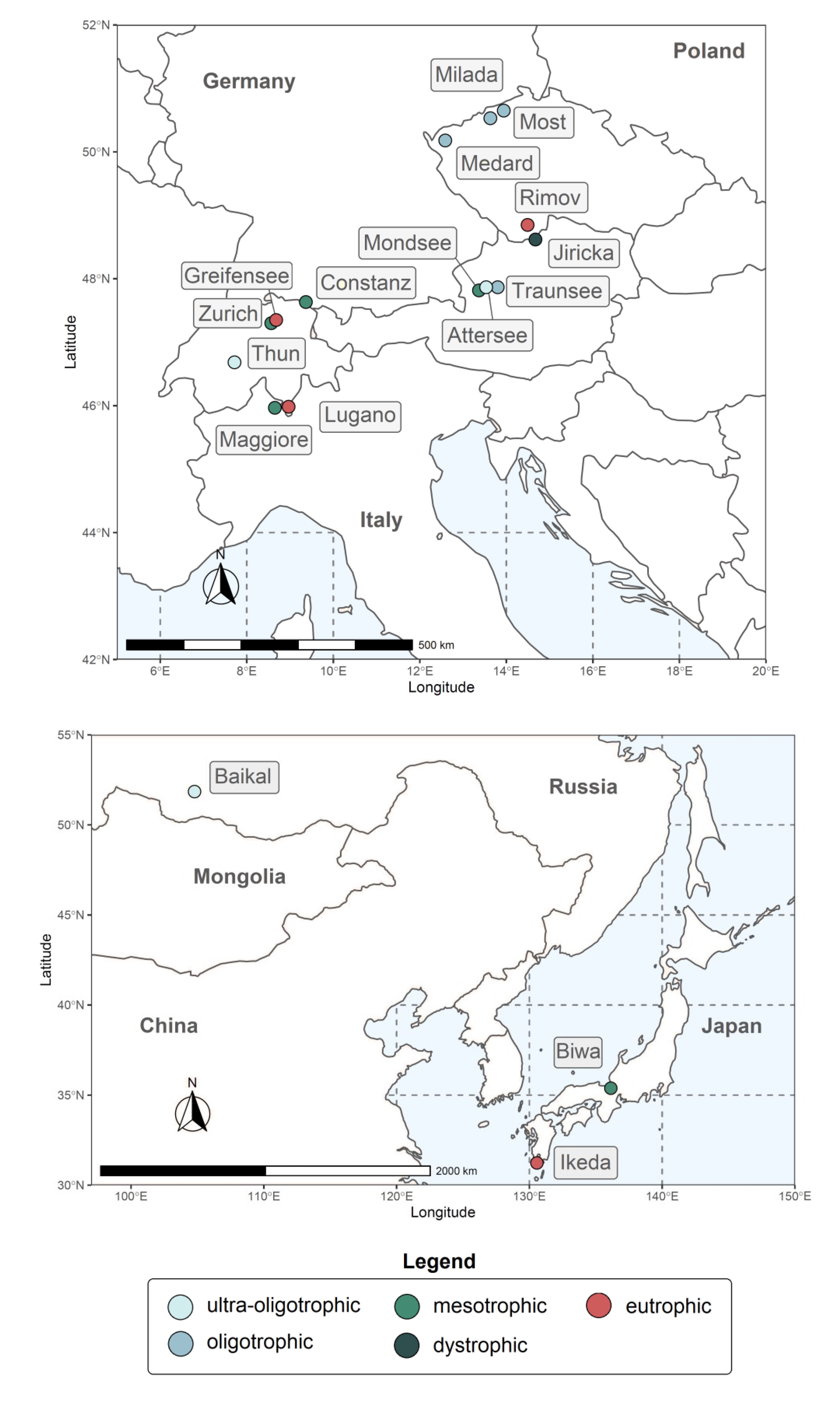
**

**Supplementary Figure S1.** Geographic location of the 17 freshwater lakes sampled in this study. Trophic state of each lake is marked by a different color.


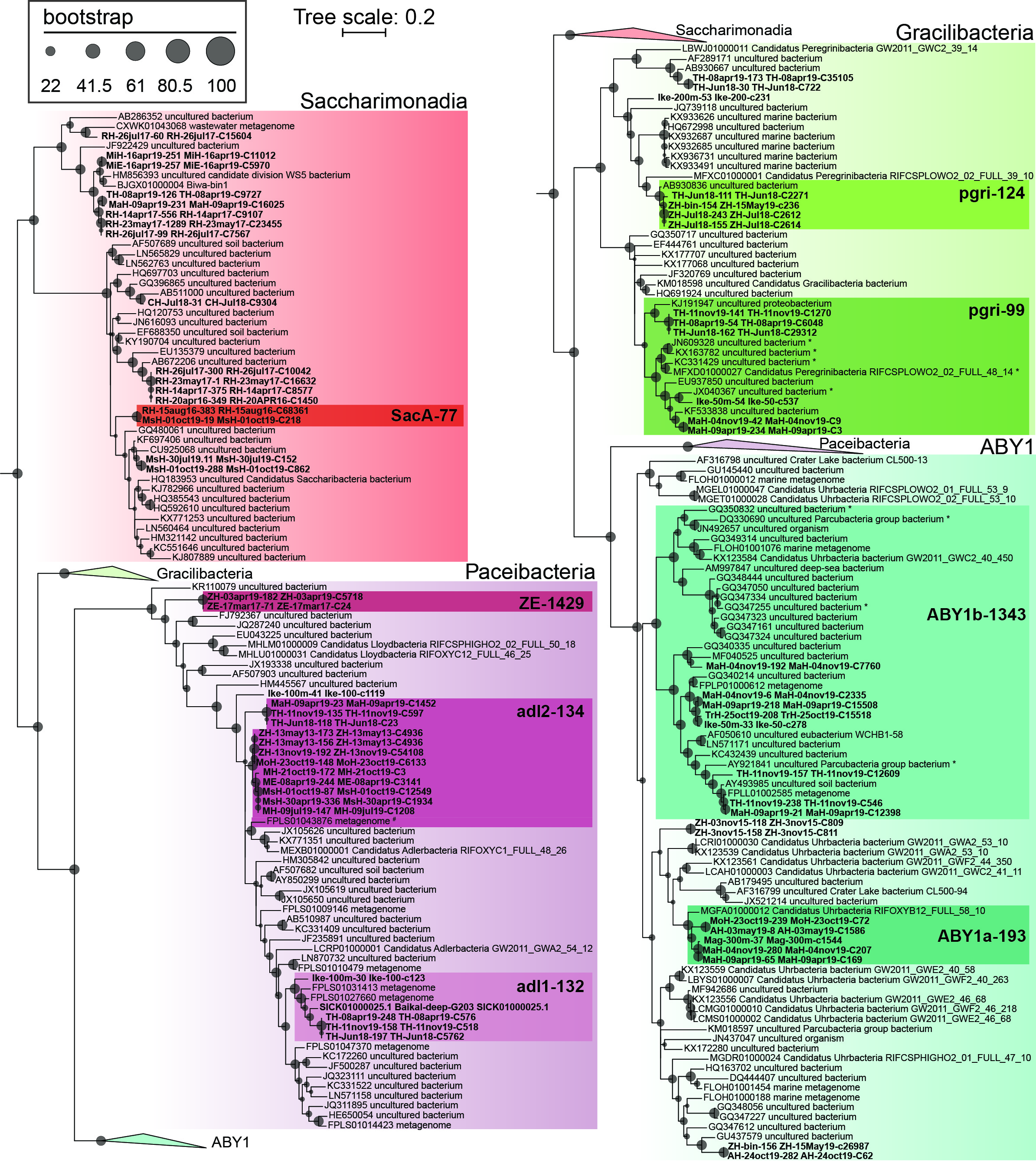
**Supplementary Figure S2.** Randomized Axelerated Maximum Likelihood (RAxML) tree of 16S rRNA genes of different classes of Patescibacteria. The tree was split in two subtrees with collapsed branches for better visibility. The 16S rRNA gene sequences recovered from MAGs generated in this study are shown in bold. Targets for eight newly designed oligonucleotide probes for CARD-FISH are indicated with different colors. Bootstrap values (100 bootstraps) are indicated as differently sized circles, the tree scale is given on top. Asterisks indicate sequences not targeted by probes pgri-99 and ABY1b-1343, # indicate one outgroup hit of probe adl2-134.


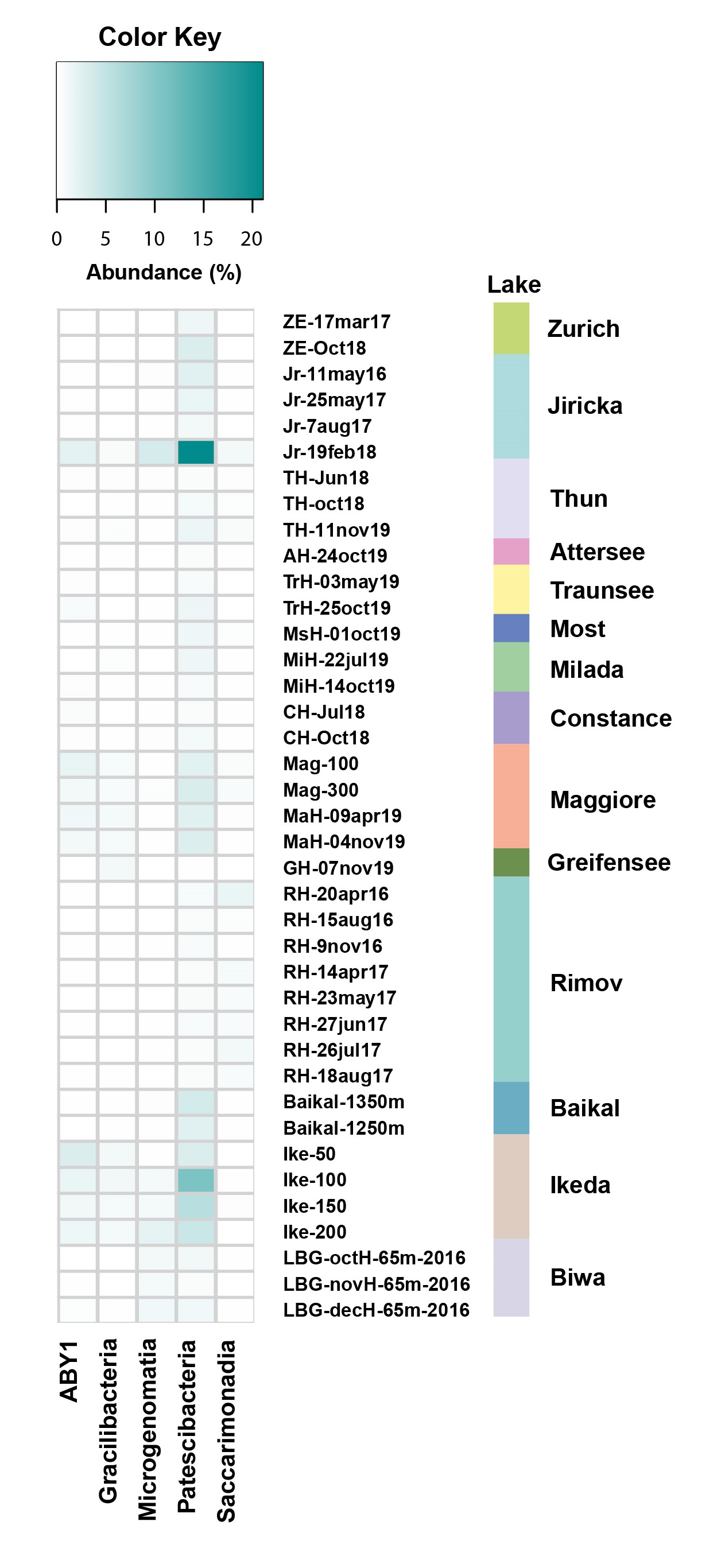
**Supplementary Figure S3.** Abundance (%) of 16S rRNA gene sequences affiliated to different classes in CPR. Samples that had < 1% of total abundance in any CPR class were not added to the plot.


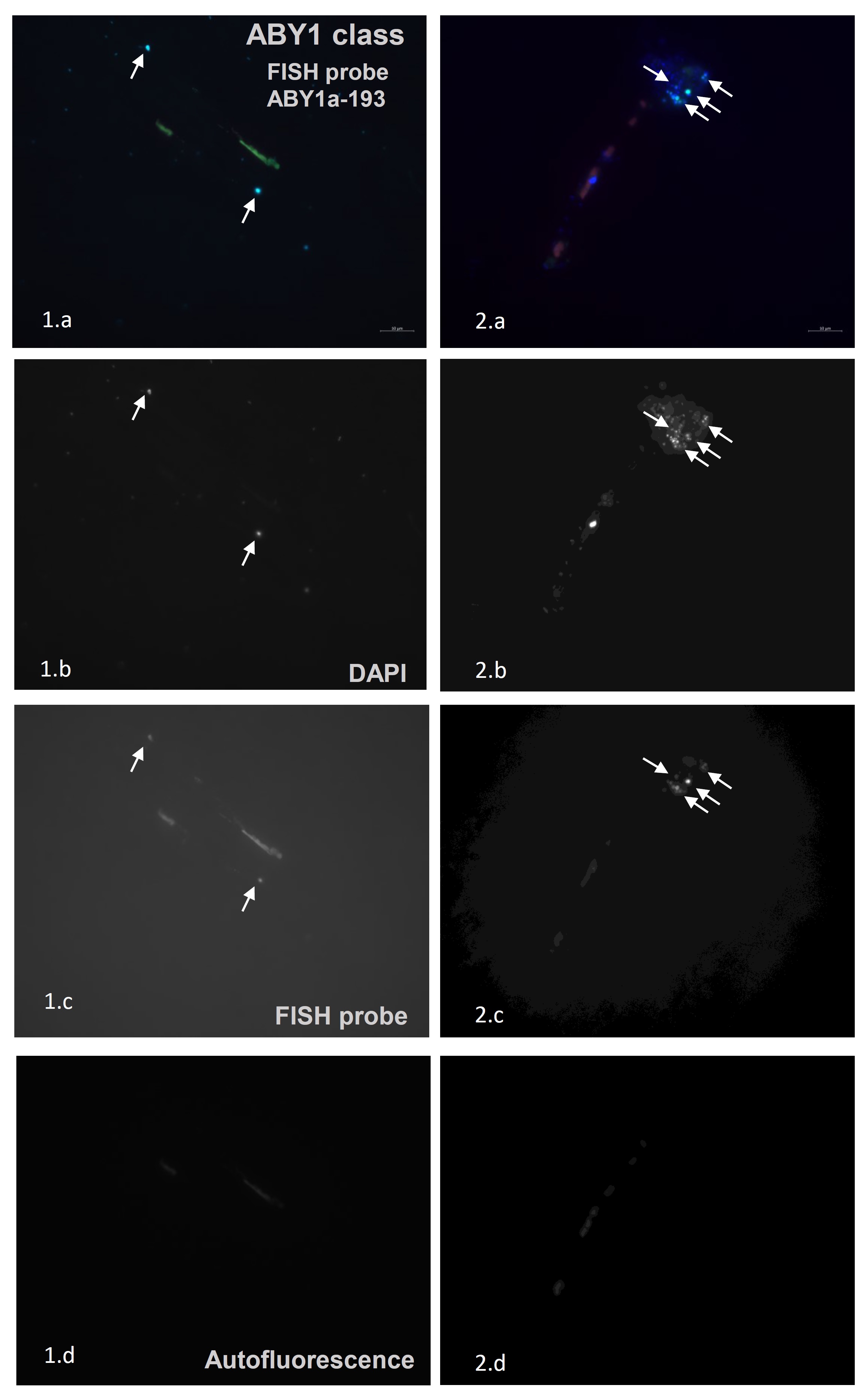
**Supplementary Figure S4.** Epifluorescence microscopy images of ABY1 class members using the ABY1a-193 probe. Panels labeled with a. are the overlap of the probe (green), DAPI (blue) and autofluorescence (red) signal. The rest of the panels represent individual signals.


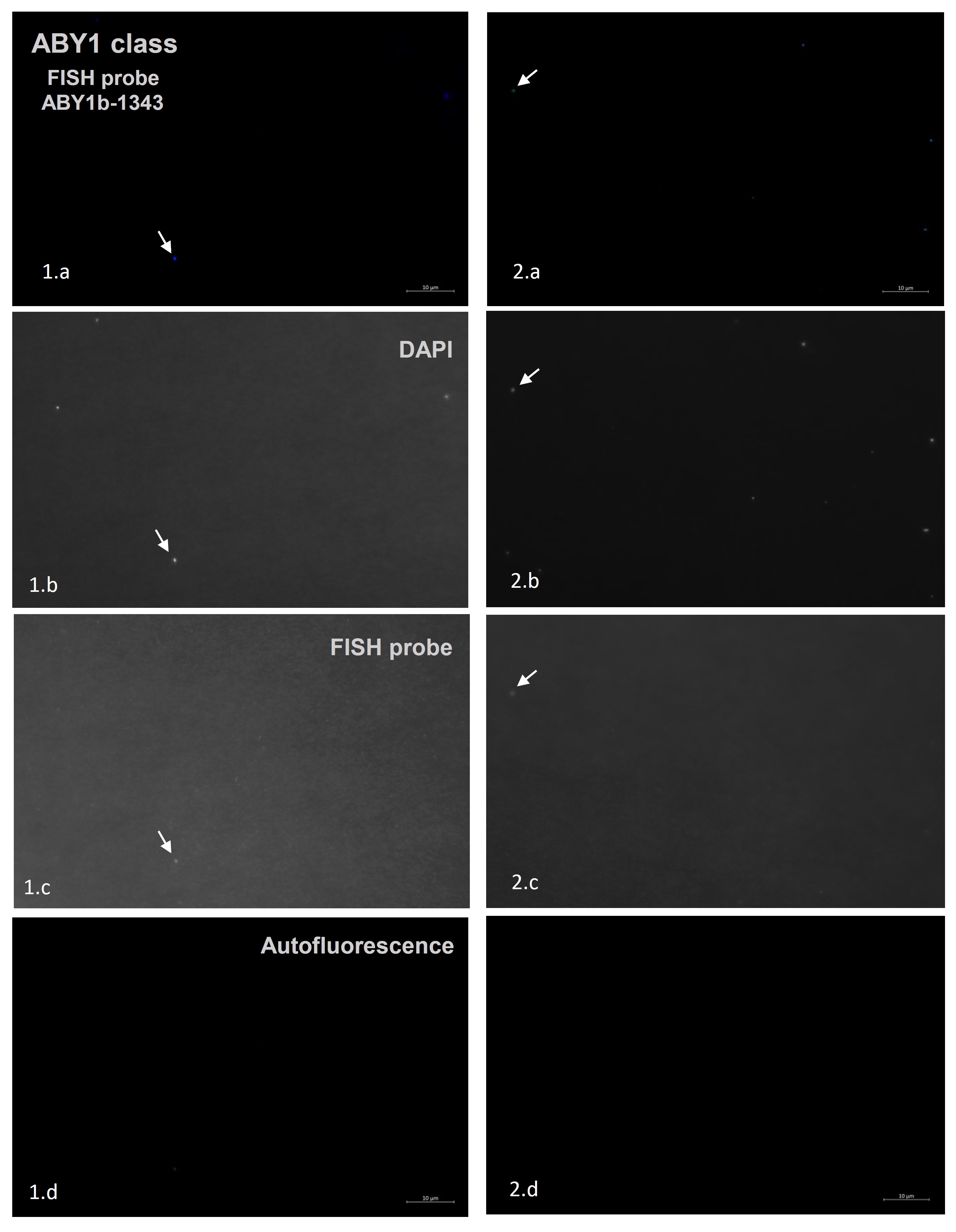
**Supplementary Figure S5.** Epifluorescence microscopy images of ABY1 class members using the ABY1b-1343 probe. Panels labeled with a. are the overlap of the probe (green), DAPI (blue) and autofluorescence (red) signal. The rest of the panels represent individual signals.


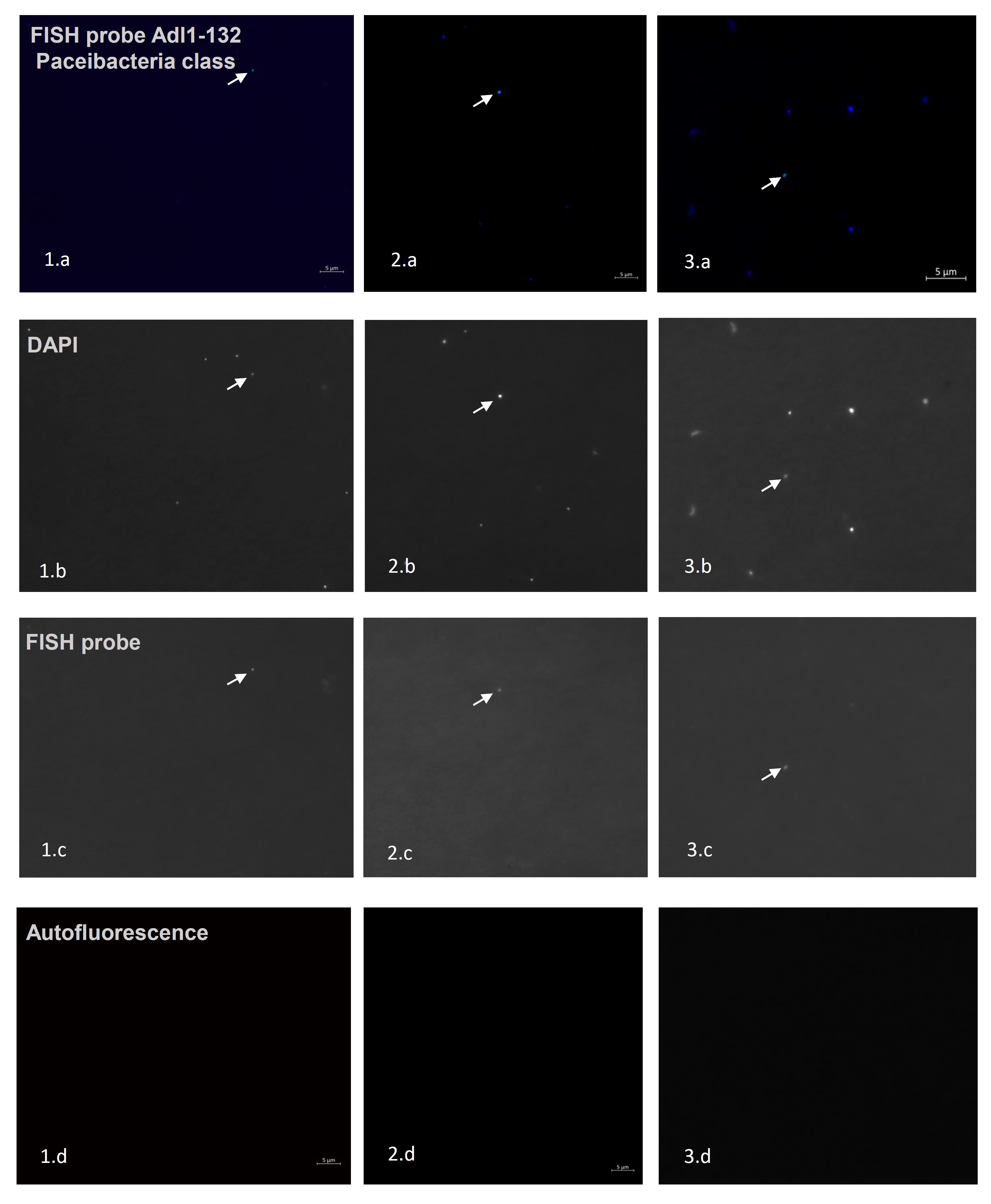
**Supplementary Figure S6.** Epifluorescence microscopy images of Paceibacteria class members using the Adl1-132 probe. Panels labeled with a. are the overlap of the probe (green), DAPI (blue) and autofluorescence (red) signal. The rest of the panels represent individual signals.


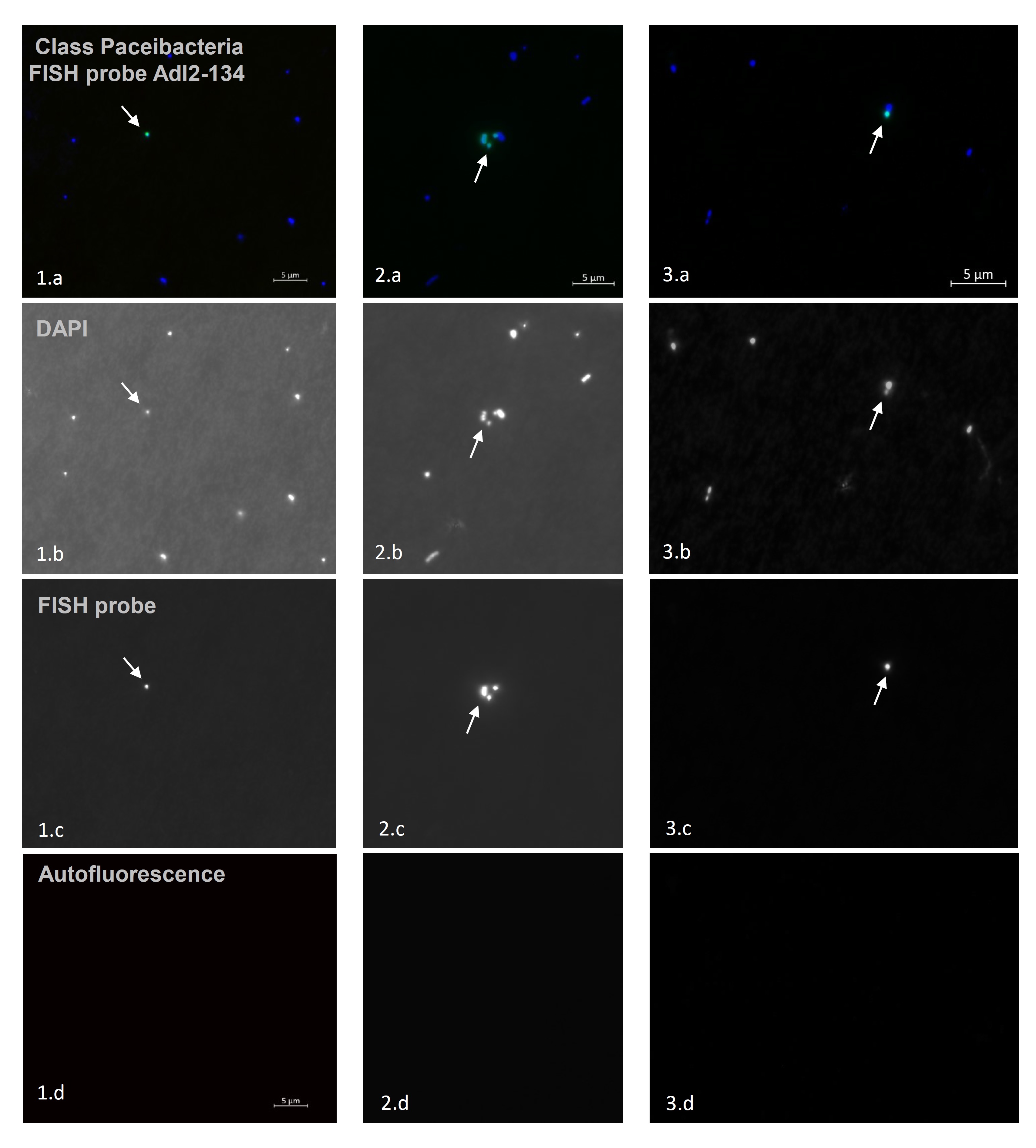
**Supplementary Figure S7A.** Epifluorescence microscopy images of Paceibacteria class members using the Adl1-134 probe. Panels labeled with a. are the overlap of the probe (green), DAPI (blue) and autofluorescence (red) signal. The rest of the panels represent individual signals.


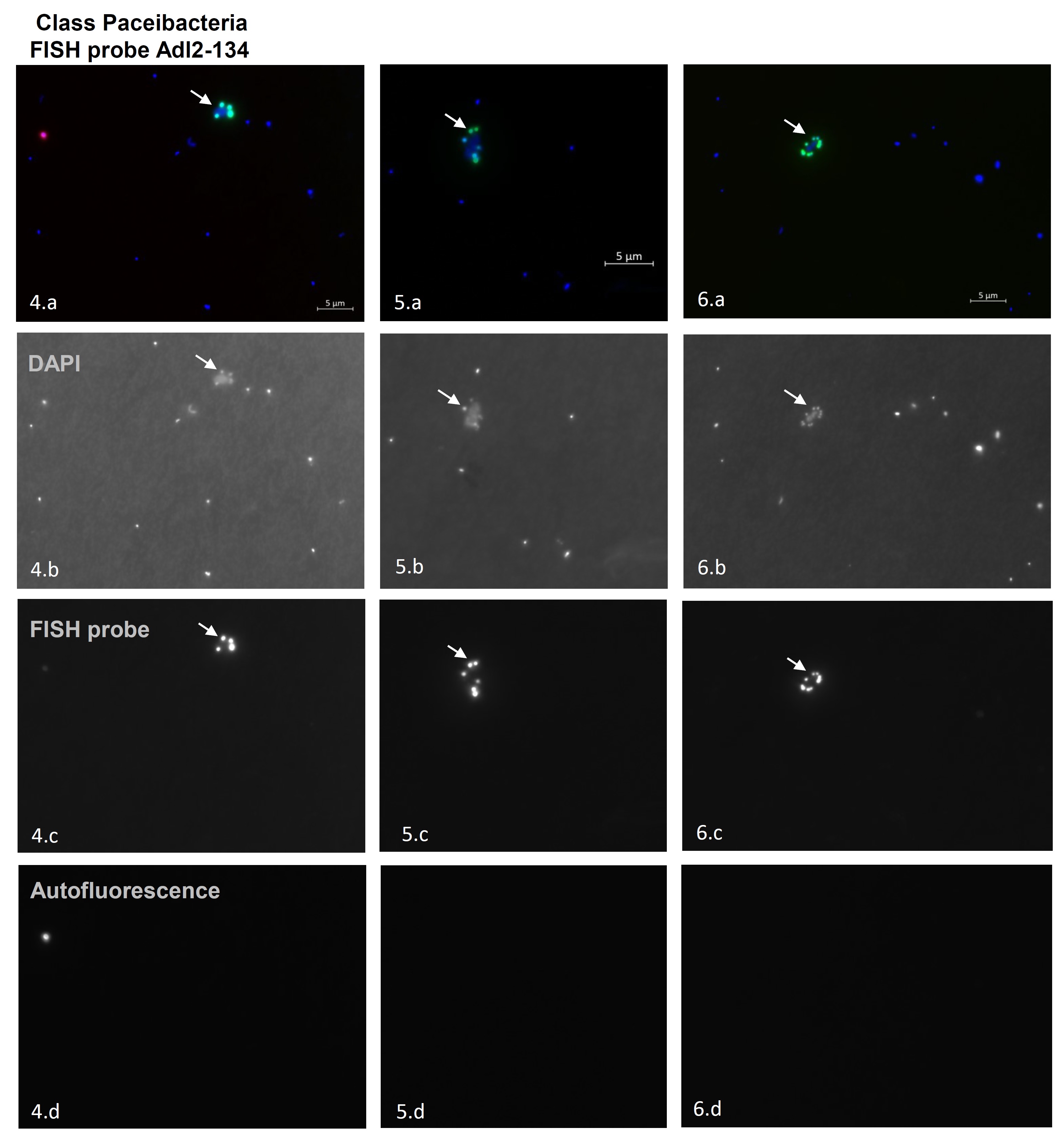
**Supplementary Figure S7B.** Epifluorescence microscopy images of Paceibacteria class members using the Adl1-134 probe. Panels labeled with a. are the overlap of the probe (green), DAPI (blue) and autofluorescence (red) signal. The rest of the panels represent individual signals.

**Supplementary Figure S8.** Epifluorescence microscopy images of Gracilibacteria class members using the Pgri-99 probe. Panels labeled with a. are the overlap of the probe (green), DAPI (blue) and autofluorescence (red) signal. The rest of the panels represent individual signals.


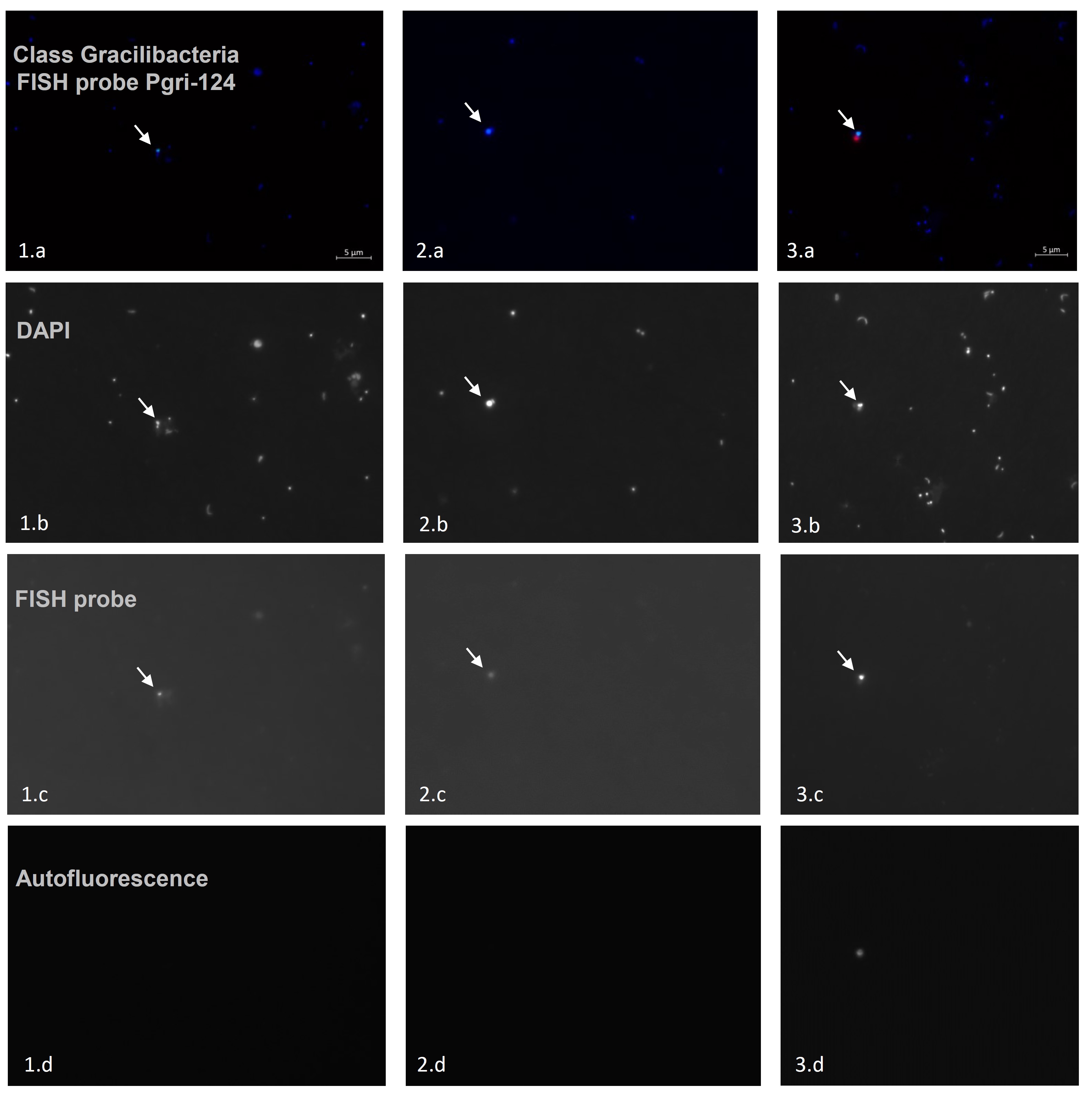
**Supplementary Figure S9.** Epifluorescence microscopy images of Gracilibacteria class members using the Pgri-124 probe. Panels labeled with a. are the overlap of the probe (green), DAPI (blue) and autofluorescence (red) signal. The rest of the panels represent individual signals.


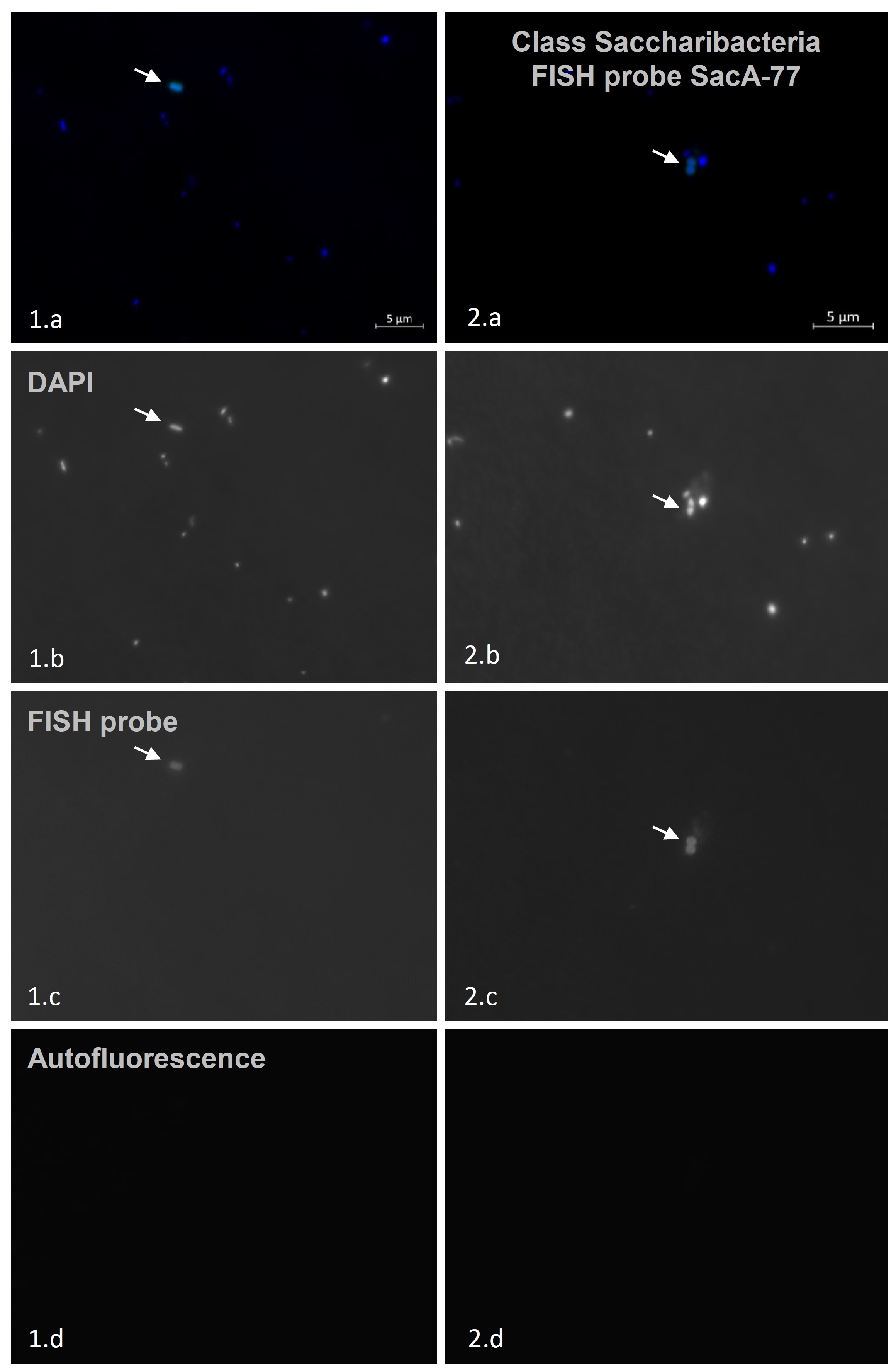
**Supplementary Figure S10.** Epifluorescence microscopy images of Saccharimonadia class members using the SacA-77 probe. Panels labeled with a. are the overlap of the probe (green), DAPI (blue) and autofluorescence (red) signal. The rest of the panels represent individual signals.


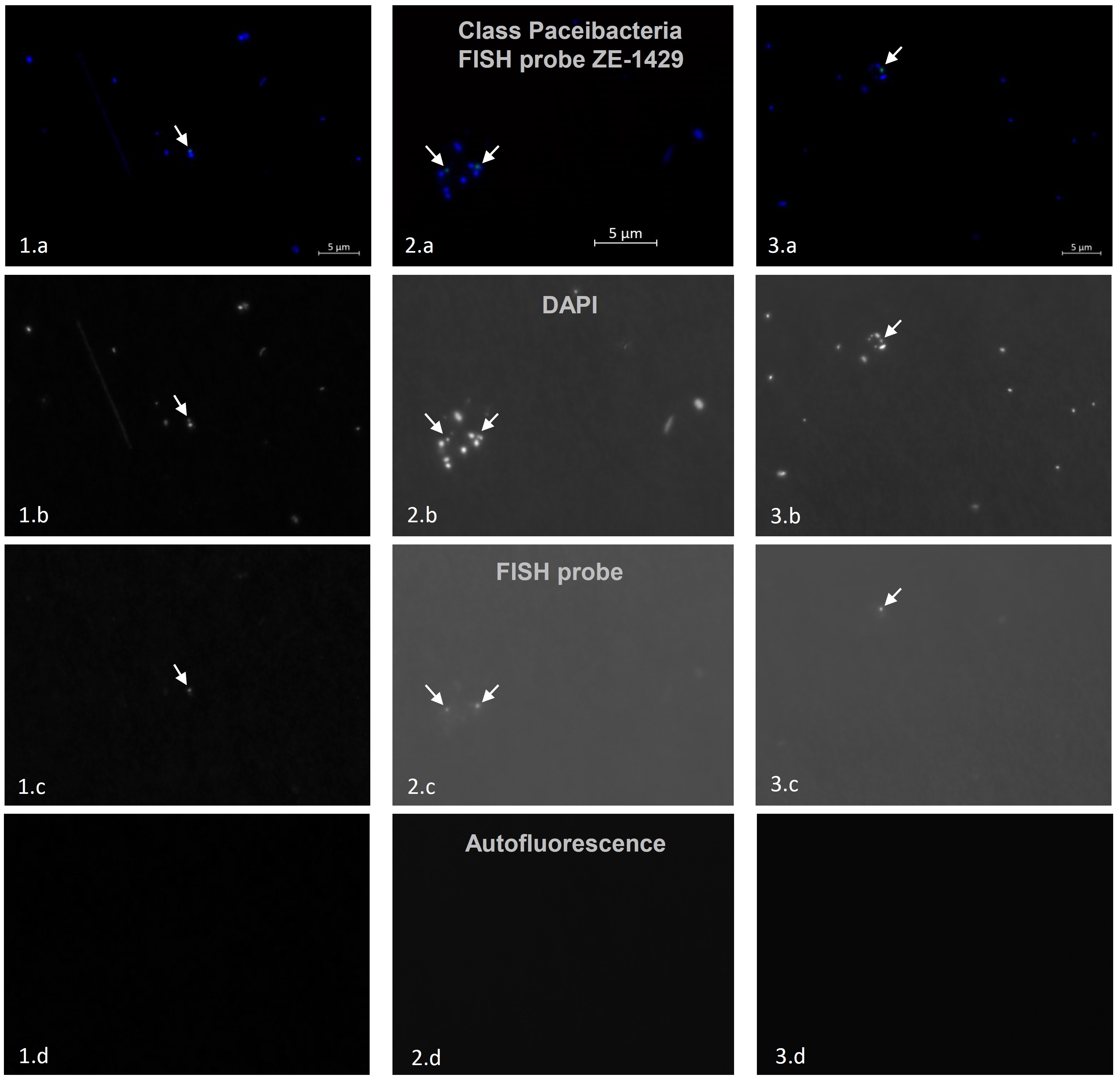
**Supplementary Figure S11.** Epifluorescence microscopy images of Paceibacteria class members using the ZE-1429 probe. Panels labeled with a. are the overlap of the probe (green), DAPI (blue) and autofluorescence (red) signal. The rest of the panels represent individual signals.


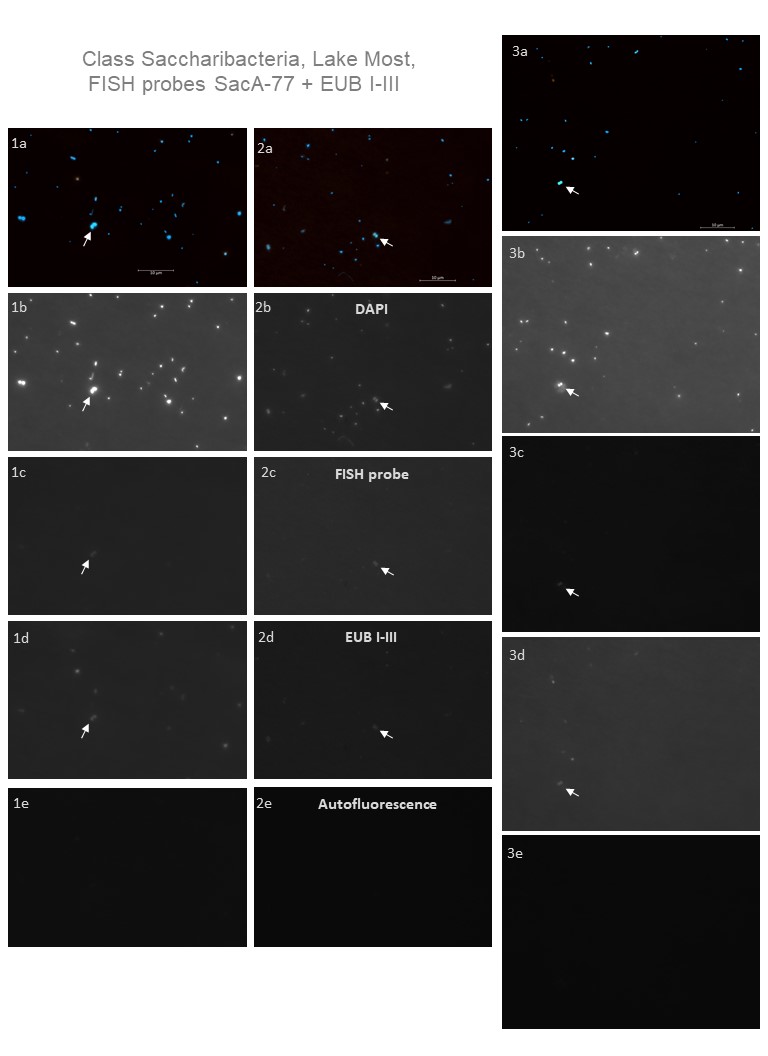
**Supplementary Figure S12.** Positive control for the Saccharibacteria class members performed using the SacA-77 and EUB I-III probes. Panels labeled with a. are the overlap of DAPI (blue), the Sac-77 probe (green), the EUB I-III probe (orange) and autofluorescence (red) signal. The rest of the panels represent individual signals.

**Supplementary Figure S13.**
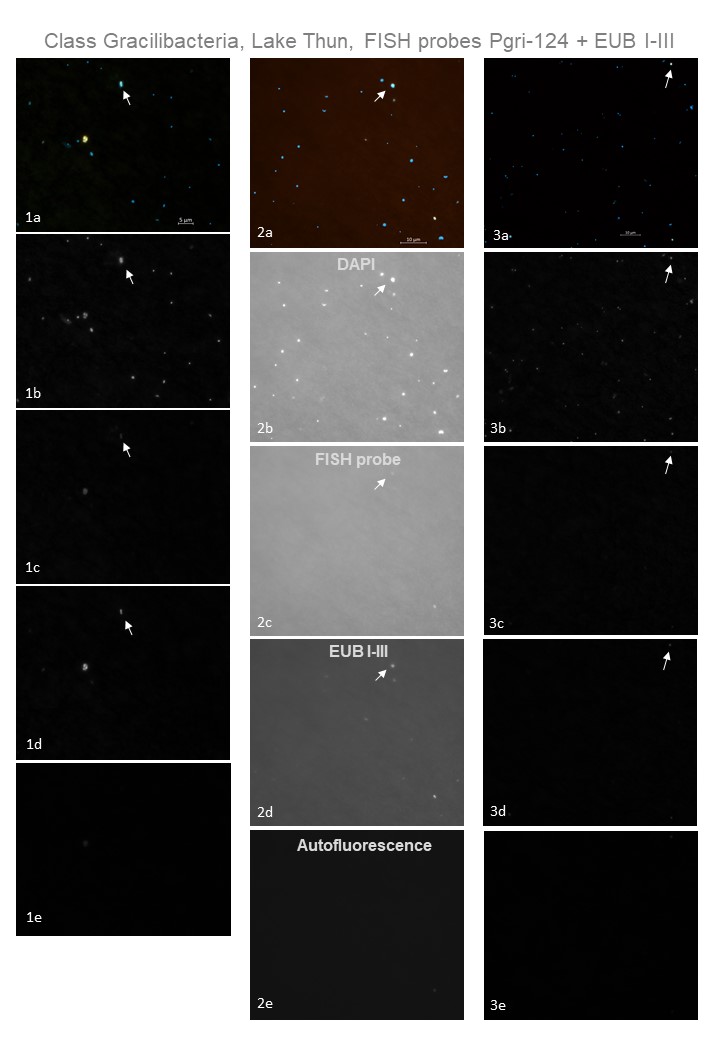
 Positive control for the Gracilibacteria class members performed using the Pgri-124 and EUB I-III probes. Panels labeled with a. are the overlap of DAPI (blue), the Pgri-124 probe (green), the EUB I-III probe (orange) and autofluorescence (red) signal. The rest of the panels represent individual signals.

**Supplementary Figure S14a.**
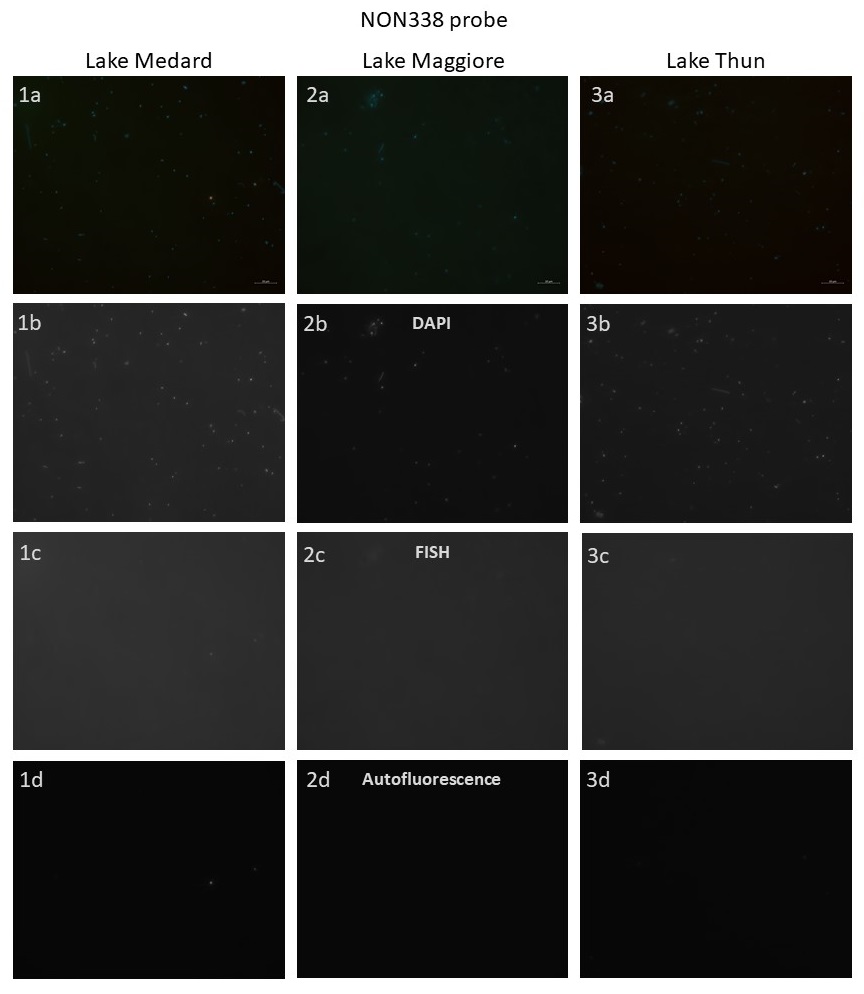
 Negative controls for filters used for FISH from different lakes. Panels labeled with a. are the overlap of DAPI (blue), the NON338 probe (green) and autofluorescence (red) signal. The rest of the panels represent individual signals.

**Supplementary Figure S14b.**
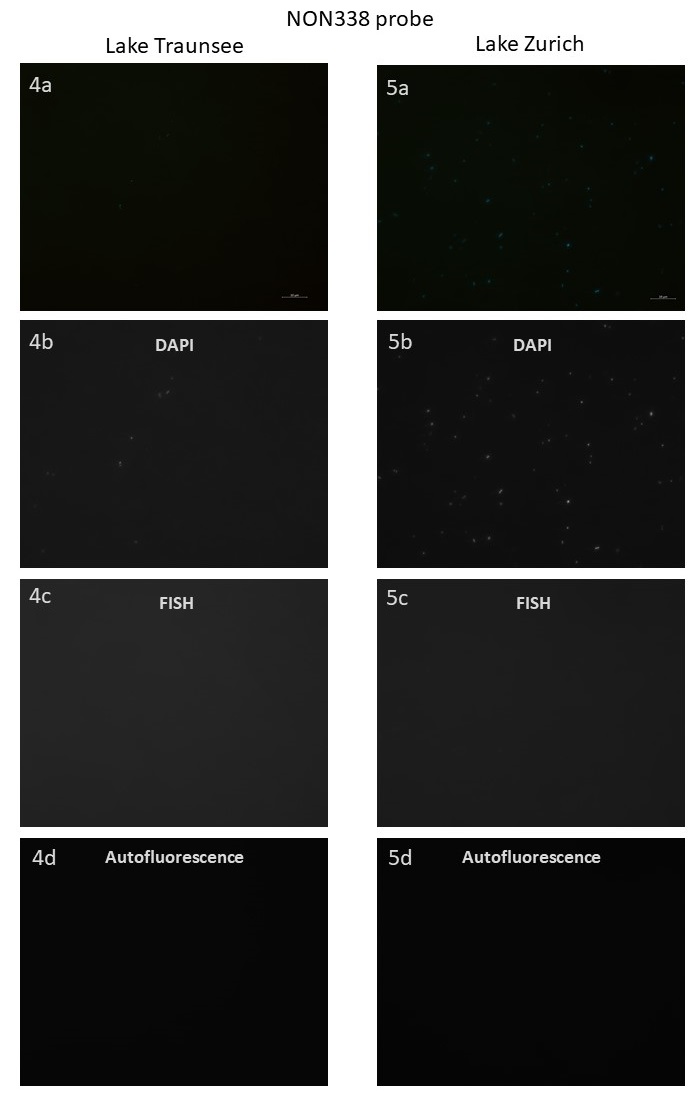
 Negative controls for filters used for FISH from different lakes. Panels labeled with a. are the overlap of DAPI (blue), the NON338 probe (green) and autofluorescence (red) signal. The rest of the panels represent individual signals.

**Supplementary Figure S15a.**
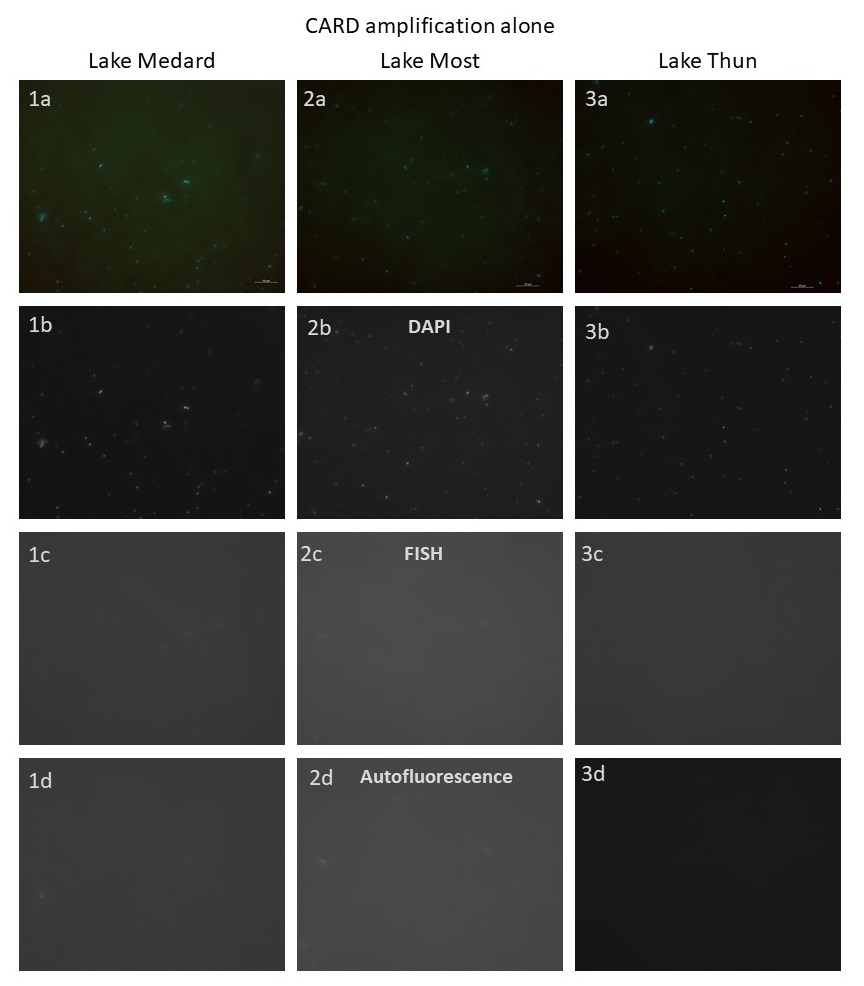
 Negative controls for filters used for FISH from different lakes. Panels labeled with a. are the overlap of DAPI (blue), CARD amplification (green) and autofluorescence (red) signal. The rest of the panels represent individual signals.


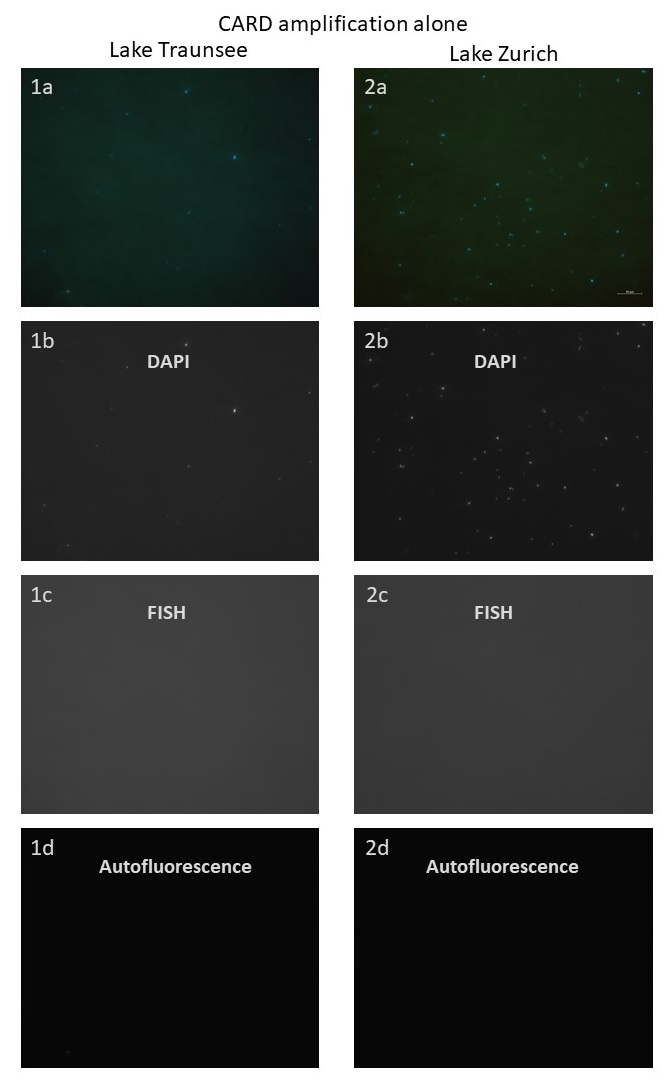
**Supplementary Figure S15b.** Negative controls for filters used for FISH from different lakes. Panels labeled with a. are the overlap of DAPI (blue), CARD amplification (green) and autofluorescence (red) signal. The rest of the panels represent individual signals.


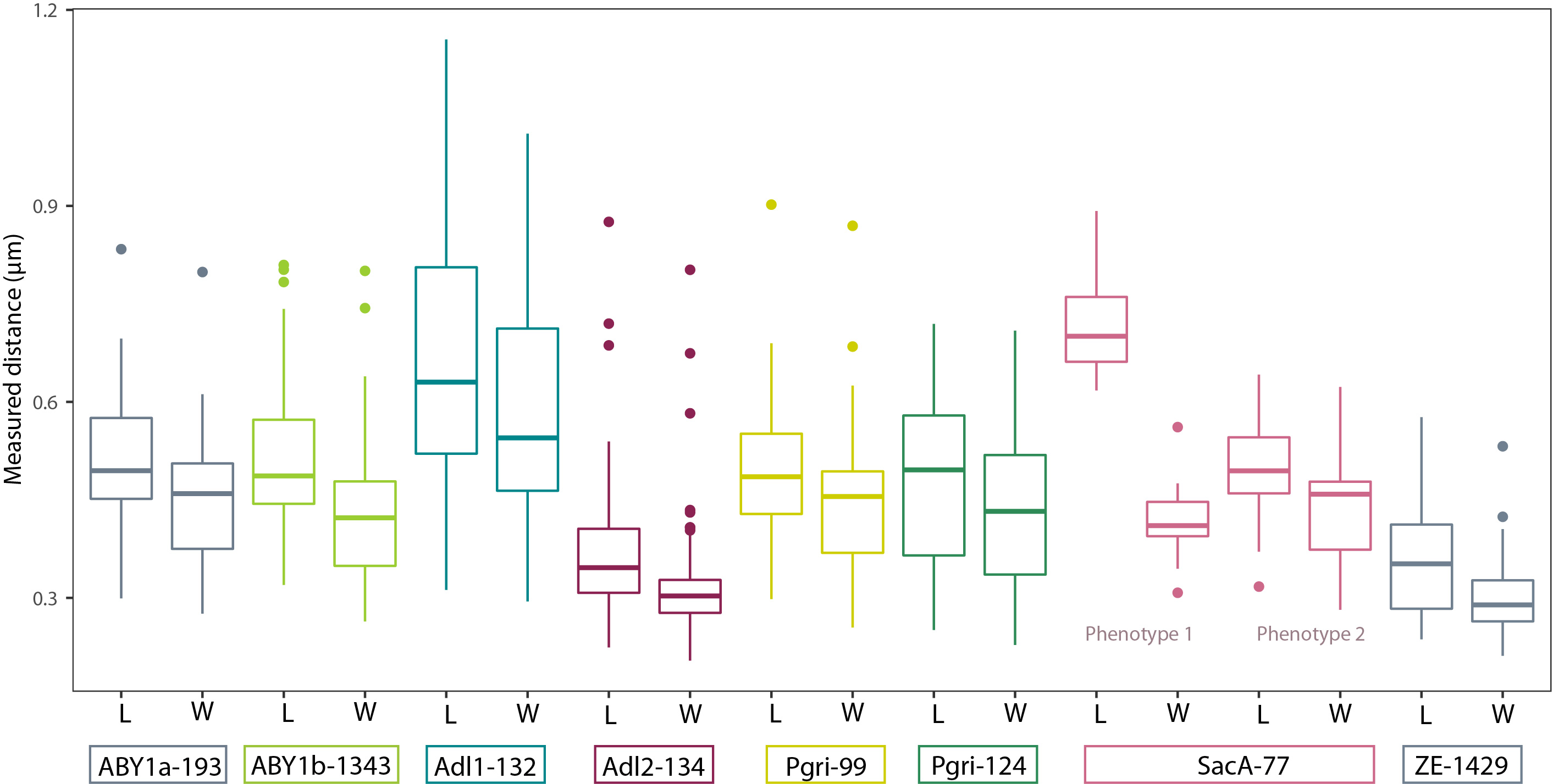
**Supplementary Figure S16.** Boxplots for the length and width of individual CPR cells observed with the 8 FISH probed designed and tested in this study. L – Length, W – Width.


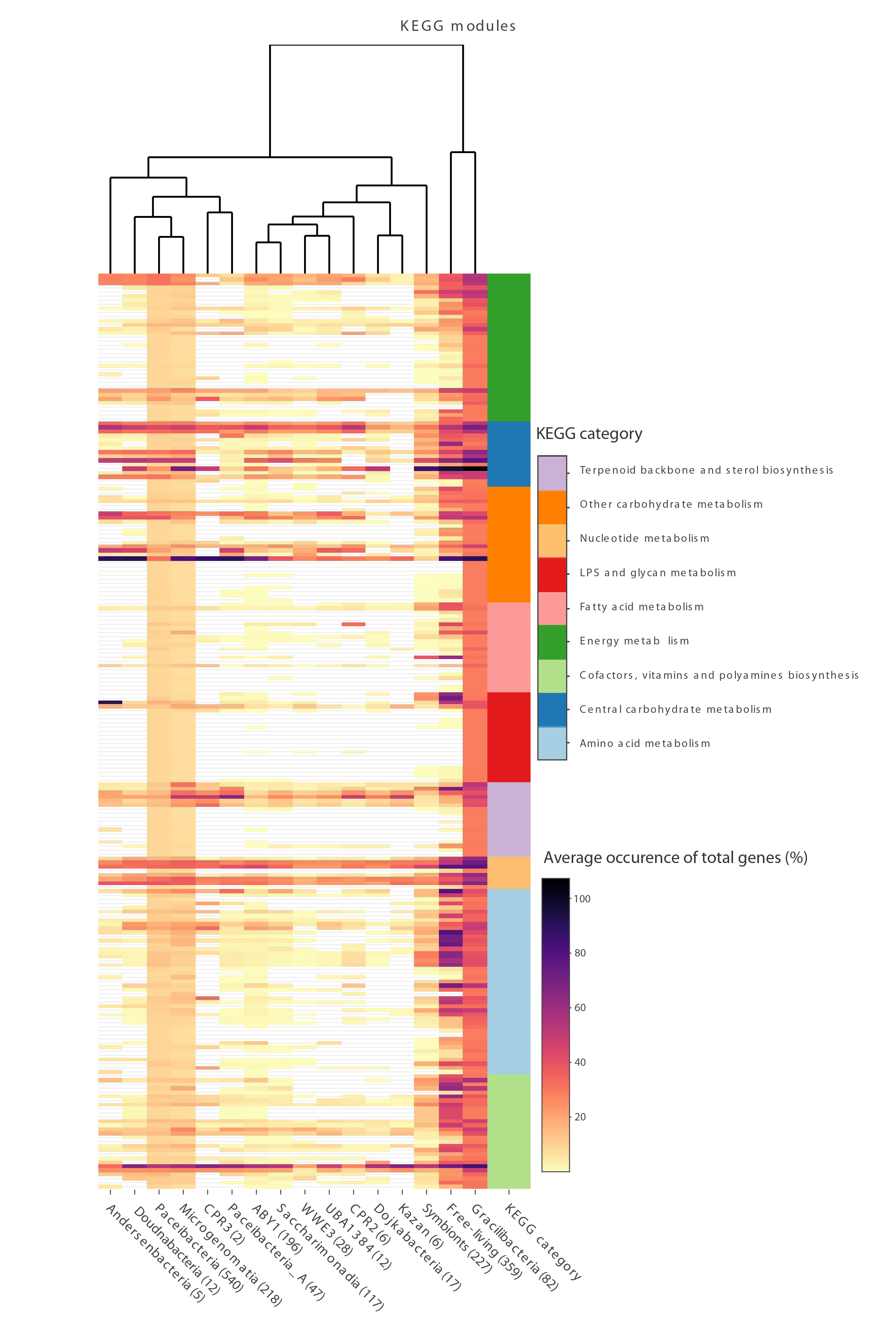
**Supplementary Figure S17.** Percentage of genes in each KEGG module encoded by CPRs, free-living freshwater bacteria and symbionts. All representative CPR genomes from GTDB (>40% completeness, <5% contamination) were included in this analysis together with our freshwater MAGS (n=1288). The same quality criteria were applied for free-living (n=359) and symbionts (n=227). First, an average per genome was computed, followed by an average for the whole group.


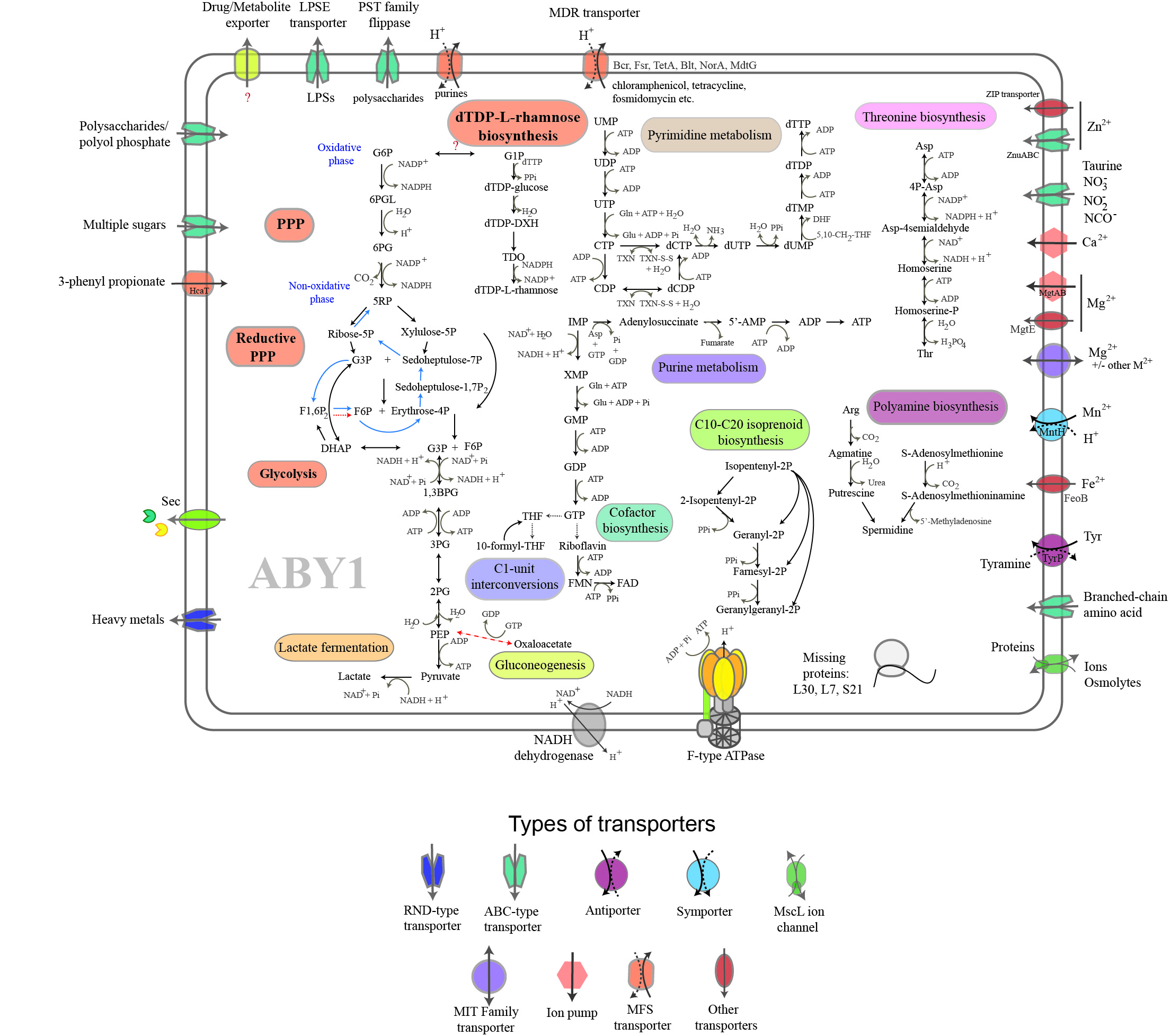
**Supplementary Figure S18.** Metabolic reconstruction of ABY1 class. Abbreviations for transporters: ABC – ATP-binding cassette, LPS – lipopolysaccharides, MDR – multi drug resistance, MFS – major facilitator superfamily, MIT – metal ion transporter, MscL – large conductance mechanosensitive ion channel, PST - polysaccharide transporter, RND – resistance-nodulation-division. Abbreviations for compounds: 1,3BPG – 1,3-bisphosphoglycerate, 2PG – 2-phosphoglycerate, 3PG – 3-phosphoglycerate, 5RP – ribulose 5-phosphate, 6PG – 6-phosphogluconate, 6PGL – 6-phoshoglucono lactone, DHAP – dihydroxyacetone phosphate, dTDP – deoxythymidine diphosphate, dTDP-DXH – dTDP-6-deoxy-D-xylo-4-hexulose, F1,6P2 – fructose 1,6-bisphosphate, F6P – fructose 6-phosphate, FAD – flavin adenine dinucleotide. FMN – flavin mononucleotide, G3P – glyceraldehyde 3-phosphate, G6P – glucose 6-phosphate, P – phosphate, PEP – phosphoenolpyruvate, PPi – pyrophosphate, TDO – dTDP-4-oxo-L-rhamnose, THF – tetrahydrofolate, TXN – thioredoxin, TXN-S-S - thioredoxin disulfide.

**Supplementary Figure S19.** Maximum likelihood phylogeny of heme-cupper oxidase subunits I-IV. a. Phylogeny of HCO subunit I (generated using 1439 sequences, 213 amino acid alignment length trimmed at maximum 50% gaps per position – the same filtering criteria was applied for all subunits). b. Phylogeny of HCO subunit II (694 sequences, 200 amino acid alignment length). c. Phylogeny of HCO subunit III (675 sequences, 156 amino acid alignment length). d. Phylogeny of HCO subunit IV (268 sequences, 76 amino acid alignment length). Bootstrap values are indicated by circle sizes.

**Supplementary Figure S20.** Maximum likelihood trees of cytochrome bd-type oxidase subunits I-II. The best selected evolutionary model was LG + F + R9 (general matrix with empirical codon frequencies counted from the data and FreeRate model with 9 categories) for both subunits. A number of 499 and 428 sequences were used for the phylogeny of subunit I and II, respectively.


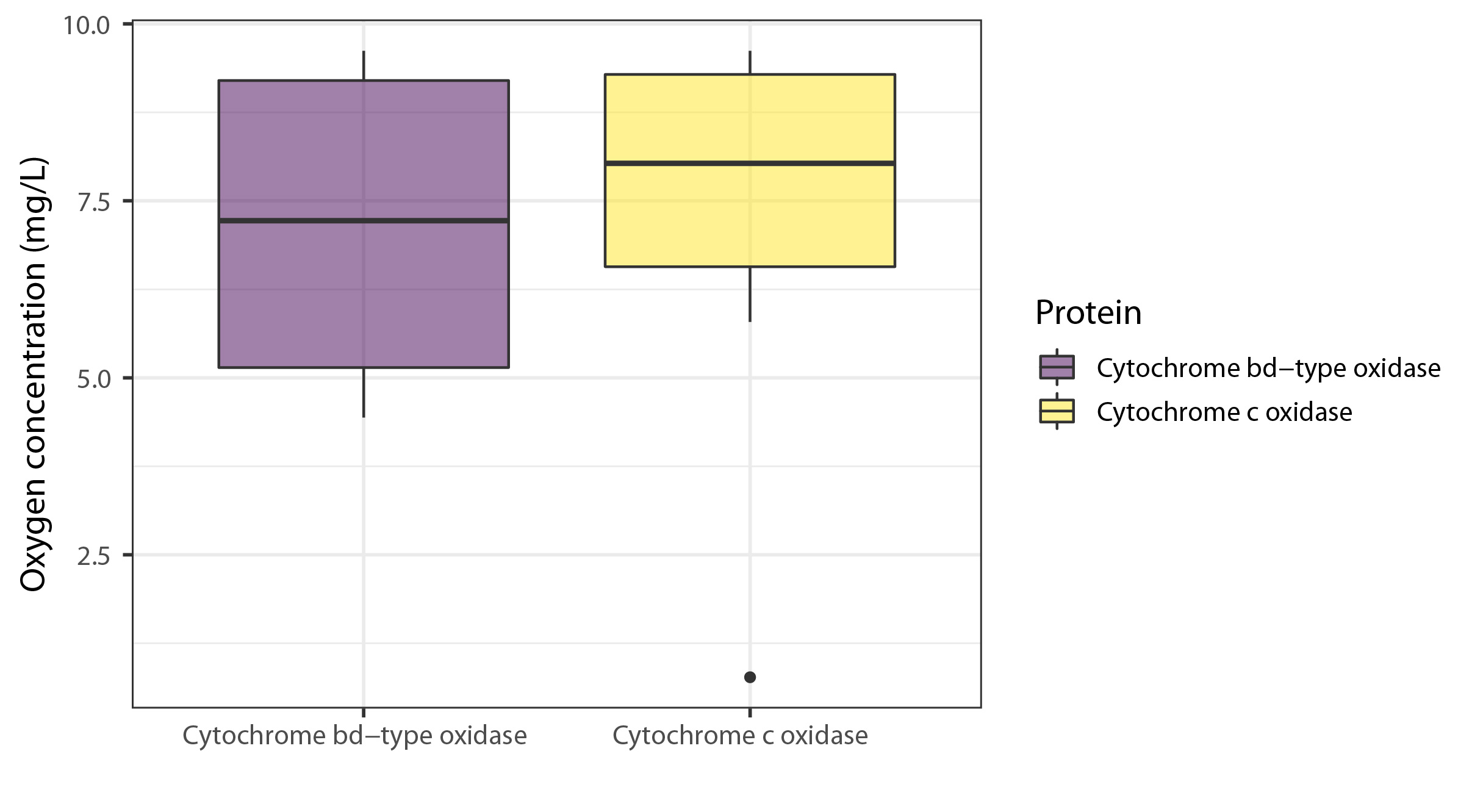


**Supplementary Figure S21.** Boxplots for oxygen concentrations measured in the lake strata from where CPR MAGs encoding terminal oxidases were obtained.


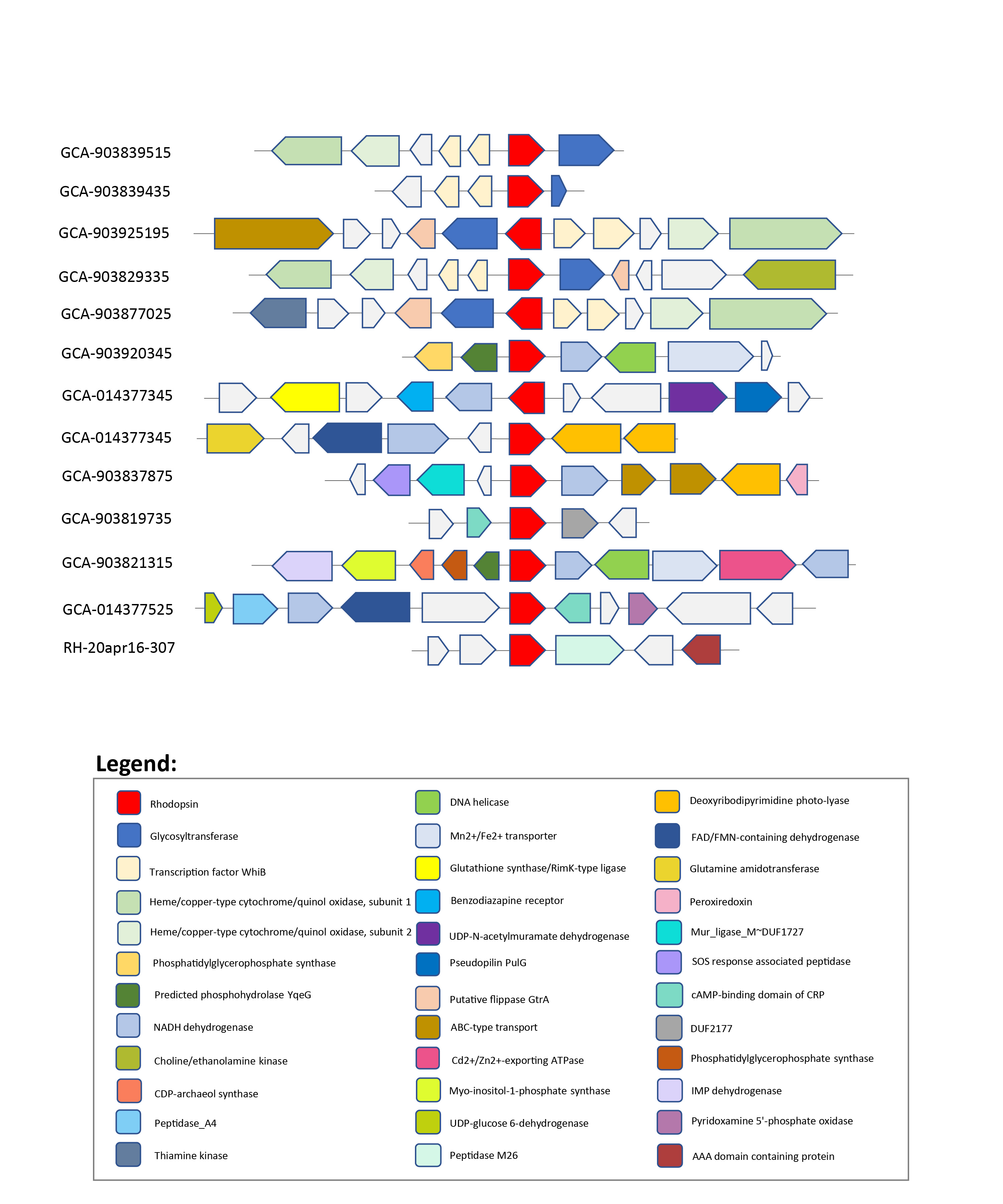
**Supplementary Figure 22.** Genomic context of predicted rhodopsins in CPRs that form a cluster in-between sensory rhodopsin I and II. Where contig length permitted, 5 genes were checked in the proximity of rhodopsins to search for putative proteins involved in the signaling transduction.
